# The Anti-Inflammatory and Curative Exponent of Probiotics: A Comprehensive and Authentic Ingredient for the Sustained Functioning of Major Human Organs

**DOI:** 10.3390/nu16040546

**Published:** 2024-02-16

**Authors:** Muhammad Safiullah Virk, Muhammad Abdulrehman Virk, Yufeng He, Tabussam Tufail, Mehak Gul, Abdul Qayum, Abdur Rehman, Arif Rashid, John-Nelson Ekumah, Xu Han, Junxia Wang, Xiaofeng Ren

**Affiliations:** 1 School of Food and Biological Engineering, Jiangsu University, Zhenjiang 212013, China; safiullahvirk@hotmail.com (M.S.V.);; 2 Department of Internal Medicine, Sheikh Zayed Hospital, Lahore 54000, Pakistan; 3 University Institute of Diet and Nutritional Sciences, The University of Lahore, Lahore 54000, Pakistan; 4 Institute of Food Physical Processing, Jiangsu University, Zhenjiang 212013, China

**Keywords:** probiotics, lactic acid bacteria, inflammation, *Lactobacillus*, *Bifidobacterium*, gut microflora

## Abstract

Several billion microorganisms reside in the gastrointestinal lumen, including viruses, bacteria, fungi, and yeast. Among them, probiotics were primarily used to cure digestive disorders such as intestinal infections and diarrhea; however, with a paradigm shift towards alleviating health through food, their importance is large. Moreover, recent studies have changed the perspective that probiotics prevent numerous ailments in the major organs. Probiotics primarily produce biologically active compounds targeting discommodious pathogens. This review demonstrates the implications of using probiotics from different genres to prevent and alleviate ailments in the primary human organs. The findings reveal that probiotics immediately activate anti-inflammatory mechanisms by producing anti-inflammatory cytokines such as interleukin (IL)-4, IL-10, IL-11, and IL-13, and hindering pro-inflammatory cytokines such as IL-1, IL-6, and TNF-α by involving regulatory T cells (Tregs) and T helper cells (Th cells). Several strains of *Lactobacillus plantarum*, *Lactobacillus rhamnosus*, *Lactobacillus casei*, *Lactobacillus reuteri*, *Bifidobacterium longum*, and *Bifidobacterium breve* have been listed among the probiotics that are excellent in alleviating various simple to complex ailments. Therefore, the importance of probiotics necessitates robust research to unveil the implications of probiotics, including the potency of strains, the optimal dosages, the combination of probiotics, their habitat in the host, the host response, and other pertinent factors.

## 1. Introduction

**Figure 1:**
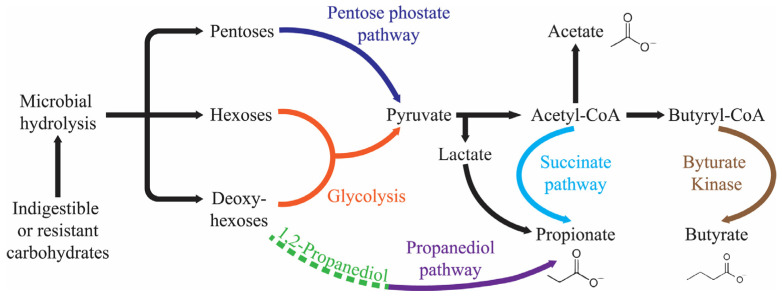
Production pathway of the SCFAs in the human gut.

The human body has an estimated count of 100 trillion microorganisms residing in the gastrointestinal lumen, more than the somatic cell count. The lumen anatomical region is the primary home for many microbial species, encompassing viruses, bacteria, fungi, and yeast [1]. The microflora residing in the gut is a significant repository of commensally existing bacteria that coexist harmoniously and perform beneficial metabolic and biological tasks for the host. Among several bacterial species, anaerobes, containing over three million different genes, as well as Firmicutes and Bacteroidetes (Gram-positive and Gram-negative, respectively) dominate the biosynthesis of short-chain fatty acids (SCFAs), i.e., butyrate, acetate, and propionate (Figure 1). Proteobacteria, Fusobacteria, Actinobacteria, and Verrucomicrobia are the other phyla that are listed as producers of SCFAs [2,3,4,5,6].

**Figure 2:**
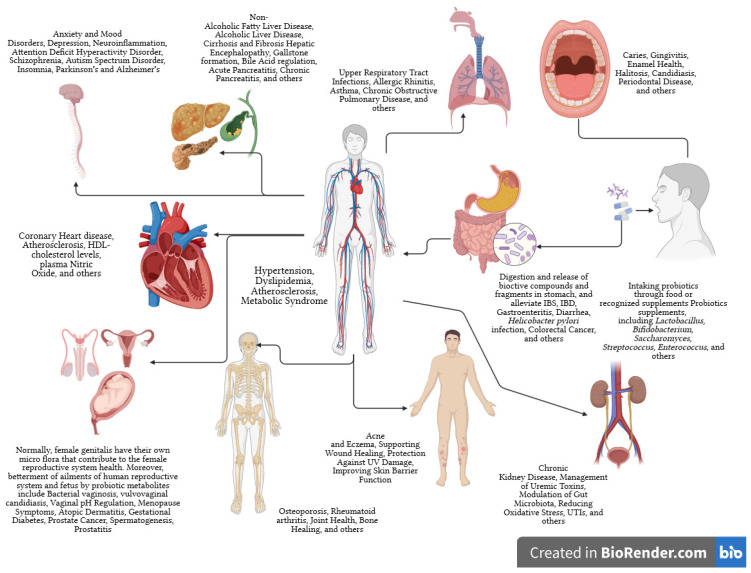
The significant health effects probiotics have on the major organs of human body. The arrows represent the flow of beneficial compounds from probiotics.

Probiotics initially treated gastrointestinal issues. They may prevent intestinal infections, aid constipation and diarrhea, improve lactose tolerance, and more [7]. The WHO and FAO define probiotics as affecting more than the intestines. Probiotics can prevent allergies, cancer, diabetes, and obesity and safeguard urogenital health (Figure 2) [8,9,10].

So far, most of the probiotics studied by researchers are gut bacteria. Probiotics’ capacity to affect the immune system and ferment in the GI tract has opened up several medicinal uses. Probiotics inhibit pathogenic germs in several ways, such as by producing bacteriocins and bioactive peptides, competing for resources, changing pH, and creating an unfavorable environment for infections. Probiotics sticking to epithelial cells prevent pathogens from interacting with surface chemicals. They or their metabolites can also interact with several epithelial cell receptors. This connection activates pro- and anti-inflammatory signaling pathways, achieving homeostasis [11,12].

The gut microbiota and its metabolites affect the heart, brain, gut, vasculature, liver, kidneys, and host immunity; however, the research subjects in this discipline require further study. The gut microbiota is complex and inter-linked. Intestinal dysbiosis in various ailments reduces metabolic activity; hence, a “one-size-fits-all” approach to treatment is ineffective. Probiotic supplementation using bacterial strains that control metabolite synthesis may help manage several ailments. Characterizing interactions between various bacterial strains is essential for finding the best probiotic bacteria and metabolites for medicinal use [13].

Prebiotics are dietary fibers metabolized by the intestinal microbiota, resulting in the modulation of the microbiota and production of SCFAs. Metabolites are produced through prebiotic fermentation, exhibiting anti-inflammatory and immunomodulatory properties, indicating their potential for therapeutic applications in various pathological conditions. Galactooligosaccharide and short- and long-chain fructans such as fructooligosaccharides and inulin have been extensively used as prebiotics, although several other dietary compounds exhibiting similar characteristics are present [14].

Synbiotics refer to combinations of probiotics and prebiotics, which can synergistically work together. Synbiotics introduced into the gastrointestinal tract promote the growth and activate the metabolism of a natural intestinal microbiota, thereby positively impacting the host’s health. Synbiotics are products where a prebiotic component specifically benefits probiotic microorganisms to enhance their survival functioning in the GI. Hence, an appropriate amalgamation of both elements in a singular product can improve the outcome compared to the efficacy of the prebiotic or probiotic individually [15].

The probiotic strain, the targeted disease or condition, and the individual all need to be considered when analyzing a probiotic supplement’s efficiency and credible findings from carefully conducted human clinical studies. The palliating effects of probiotics on different organs are overviewed in Figure 2. The transnational agreement is that probiotic species help the host in many ways, such as contesting pathogenic microorganisms for adhesion sites and nutrition, improving the epithelial lining’s barrier function, modifying the immune system, and impacting other organs through neurotransmitter synthesis, and immune system modulation [16]. Probiotics increase the production of butyrate, a vital compound for eubiosis and human health. Beneficial microorganisms modulate an intestinal environment, optimizing nutrition absorption [17].

Previously, the alleviating effects of probiotics were elaborated on different organs separately, with efforts to demonstrate probiotics’ curative exponents on various organs simultaneously being rare. Therefore, this review seeks to establish the unquestionable advantages of probiotic therapies and their effects on critical bodily systems and organs and pursue the best-performing probiotics species involved in alleviating several ailments. Moreover, this review also aims to unveil the verity of the involvement of probiotics in soothing several organs simultaneously.

## 2. Probiotics Palliate Ailments in the Oral Cavity

Extensive studies have recently examined the deployment of probiotics to treat oral diseases and preserve oral health. The genera *Lactobacillus*, *Streptococcus*, *Weissella*, *Bifidobacterium*, as well as a few dispersed species like *Saccharomyces cerevisiae* and *Bacillus subtilis*, have been shown to have high concentrations of probiotics that benefit oral health. Many strains of the oral cavity-isolated microbes, *Lactobacillus reuteri*, *Streptococcus salivarius*, *Lactobacillus brevis*, and others, have been commercially created as probiotics that promote oral health [18,19]. The species regarded as probiotics have been shown to improve the symptoms of common oral disorders, including halitosis, dental caries, oral candida infection, and periodontal disease [20,21,22].

### 2.1. Periodontal Disorders and The Use of Probiotics to Alleviate Them

The most common periodontal disorders, gingivitis and periodontitis, are chronic inflammatory diseases that erode the gum- and teeth-supporting bone. Gingivitis, a moderate periodontal condition, can lead to periodontitis, which can lead to the loosening or loss of teeth. As of 2019, 1.1 billion people worldwide have severe periodontitis, the prevalence of which has increased by 8.44% since the early 1990s [23].

**Figure 3:**
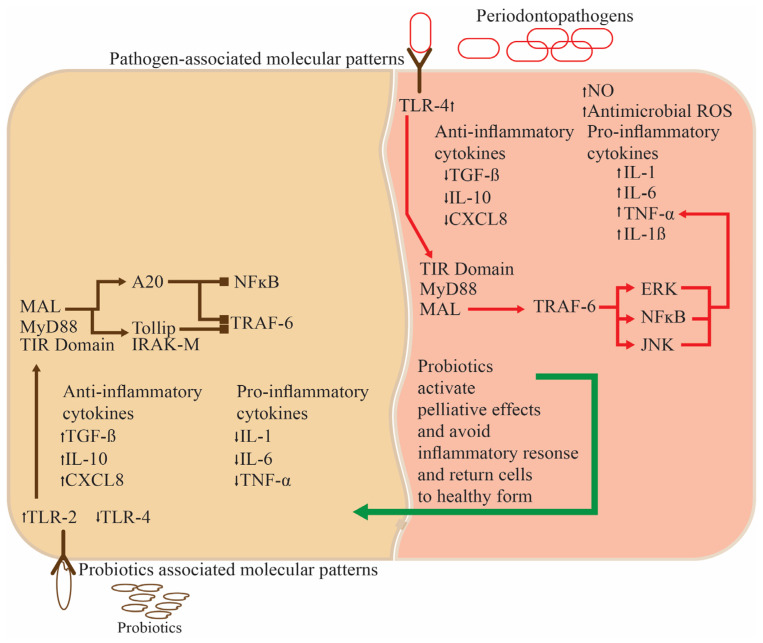
The functioning of probiotics in the oral cavity for normal functioning and anti-inflammatory activity.

Probiotic usage limits bacteria development better than non-probiotic toothpaste, avoiding periodontal (Figure 3) and dental cavities. Toothpaste lingers on the tooth’s surface for longer, and the gingival sulcus can be easily reached using a toothbrush.

Many studies have examined probiotics’ immediate and long-term effects in periodontal non-surgical treatment. Ingesting yogurt containing *Bifidobacterium animalis* subsp. lactis DN-173010 and leaving the teeth unbrushed for five days can have soothing effects. Several parameters, including gingival crevicular fluid (GCF), the gingival index (GI), bleeding on probing (BOP), the plaque index (PI), periodontal pocket depth (PPD) volume, and GCF interleukin-1 (IL-1) concentration and quantity, showed significant betterment in the patients ingesting probiotics than the placebo, showing that probiotics improve gingival inflammatory parameters and plaque accumulation even after short-term use even during missing oral hygiene measures (Table 1) [24]. Intaking probiotics other than toothpaste is also beneficial for oral health. Chronic periodontitis patients receiving *Lactobacillus reuteri* lozenge had significantly better PI, GI, BOP, and PPD than those who received the placebo. Metalloproteinases-1 (TIMP-1) and matrix metalloproteinase-8 (MMP-8) levels in GCF also differed significantly [20].

### 2.2. Streptococcus and Lactobacillus spp. as Therapeutic Agents against Dental Caries

Dental caries is a bacterially caused, multifactorial illness that causes acid demineralization of tooth enamel [25]. According to the US Surgeon General, dental caries is a chronic illness that often affects children. Three main factors affect dental caries: carbohydrate consumption, especially sugar, Streptococcus mutans infection, and immune system responses [26,27].

*Streptococcus thermophilus* and *Lactobacillus lactis* spp. lactis, which are among the 23 major dairy sector strains, can form a biofilm on hydroxyapatite and inhibit the growth of cariogenic *Streptococcus sobrinus*. *Weissella cibaria* isolates can decrease *S. mutans’* biofilm production in vitro and in vivo and hinder its proliferation [28,29]. *Lactobacillus casei* and one strain of *Lactobacillus rhamnosus* inhibit *S. mutans* and *Streptococcus sobrinus* growth in vitro. Moreover, *Streptococcus thermophiles*, *L. reuteri*, and *Lactobacillus bulgaricus* in yogurt hinder the prevalence of streptococci, especially *S. mutans* (Table 1). Hence, the regular use of probiotic-rich dairy products, including yogurt, milk, and cheese, reduces dental plaque and salivary cariogenic streptococci [30,31,32].

A statistically significant decrease in *S. mutans* was detected in a test group that received *L. reuteri*, and the impact persisted for at least 21 days. The effectiveness of mixed cultures against oral bacteria appears to be limited. The efficacy of the probiotic strain *L. reuteri* found in Indian curd in reducing the presence of salivary *S. mutans* is evident. Furthermore, this effect has been observed to persist for a certain duration following administration [33].

### 2.3. Bifidobacterium and Lactobacillus spp. Curing Gingivitis

Patients with mild to severe gingivitis receiving *L. reuteri* formulations at moderate doses exhibit a significantly decreased GI. *L. reuteri* also significantly reduces plaque and gingivitis in patients (Table 1) [34].

Several studies have observed the clinical effectiveness of several probiotics, such as *Bifidobacterium animalis*, *Bacillus* species, and *L. reuteri*, in treating gingivitis [35,36,37]. Several trials have shown that probiotics reduce gingival indices, bleeding, and plaque. A recent RCT found that a once-daily dose of *B. animalis*-enriched yogurt reduced plaque and bleeding scores compared to the people taking plain yogurt [24].

### 2.4. Halitosis and Its Establishment and Rectification by Probiotics

Volatile molecules arising from pathological or non-pathological oral or non-oral sources combine to generate halitosis. These volatile substances include short-chain fatty acids, amines, phenyl compounds or alcohols, ketones, aliphatic compounds, sulfur compounds, nitrogen-containing chemicals, and aromatic compounds [38,39]. Anaerobic bacteria cause halitosis by breaking down salivary and dietary proteins to produce amino acids, which are converted into volatile compounds such as methanethiol and hydrogen sulfide [40]. Different strains of *W. cibaria* can prevent *Fusobacterium nucleatum* from producing volatile sulfur compounds by synthesizing hydrogen peroxide and preventing *F. nucleatum* from proliferating (Table 1). Additionally, gargling with a *W. cibaria*-enriched solution causes a decrease in the generation of methanethiol and hydrogen sulfide, reducing foul breath [29].

#### 2.4.1. *Streptococcus salivarius*: An Efficacious Element against Halitosis

*Streptococcus salivarius* is the most common commensal probiotic in the mouths of people without halitosis. *S. salivarius* produces bacteriocins which reduce the population of volatile sulfur compound-producing bacteria [41]. The administration of gum or lozenges containing *S. salivarius* K12 resulted in a decrease in volatile sulfur compounds in individuals with halitosis (Table 1) [42,43]. By establishing a healthy tongue microbial ecology, probiotics that protect periodontal health may reduce halitosis. In oral health, the dorsal posterior surface of the tongue near the circumvallate papillae is home to a large population of Gram-negative bacteria that cause bad breath. However, assessing and maintaining oral hygiene in these places is tricky [44]. The tongue is sometimes considered more prominent than the periodontal recesses in terms of the species that inhabit it. Specific adaptation to each recess is important, and probiotic strains meant to colonize periodontal recesses may not colonize the tongue and improve dental health [45,46].

#### 2.4.2. *Streptococcus salivarius*’s Enmity against *Streptococcus pyogenes*—An Oral Pathogen

*Streptococcus pyogenes*, a major bacterial pathogen, predominantly affects humans and causes moderate localized infections to severe invasive infections with potentially deadly results. Acute rheumatic fever and poststreptococcal glomerulonephritis can result from ineffective *S. pyogenes* treatment. This pathogen also causes invasive infections, including necrotizing fasciitis and toxic shock syndrome, which cause high morbidity and mortality [47].

*S. pyogenes* causes non-bullous impetigo, a common childhood skin disease. The pruritic erythematous rash usually starts in the perioral or perinasal area and progresses to vesicular lesions. Blisters often burst and form a honey-colored crust. The face and lower extremities are particularly affected by highly localized lesions. Impetigo rarely causes systemic symptoms [47]. Two salivaricin-producing *Streptococcus salivarius* strains, 20P3 and 5, given to children via milk supplementation, were shown to exhibit higher colonization levels. After drinking milk enriched with *S. salivarius*, the children experienced a significant increase in SalA-like inhibitory activity in their indigenous streptococcal tongue populations. *S. salivarius* (SalA producer) made up fewer than 5% of the tongue bacteria. After drinking *S. salivarius*-supplemented milk, the children’s tongues contained more SalA synthesizers and had increased inhibitory activity. *S. salivarius* probiotics, which produce SalA, may persistently boost SalA-dependent protection against *S. pyogenes* infections. These outcomes signify that SalA strongly diminishes *S. pyogenes*, making this noteworthy [48].

### 2.5. Lactobacillus spp. as pH and Saliva Regulators in the Oral Cavity

Probiotic-treated dairy products elevate salivary pH significantly (Table 1). This notion is consistent with clinical trials showing that probiotic-containing yogurt and curd improve salivary pH. Probiotic consumption increases pH levels because probiotic bacteria compete with other microorganisms to reduce their numbers. Therefore, salivary pH rises when acidogenic bacteria decrease and acid production decreases. Due to the close relationship with pH imbalances, these fluctuations in pH affect the control of dental caries. In curd with probiotics, salivary pH increased compared to a curd lacking them, exhibiting that added probiotics cause salivary pH to rise (Table 1) [49,50].

Probiotics increase saliva production in edentulous patients, which helps xerostomia/hyposalivation. Patients receiving regular probiotics have significantly increased saliva volume and moderately changing saliva pH [51]. Probiotic strains change saliva’s immunoglobulins and mucins, according to animal research. Another finding of that experiment was the positive influence of probiotics on hyposalivation sufferers [52].

Probiotics, a type of commensal bacteria, effectively increase oral epithelial cell beta defensin-2 (BD-2) expression [53]. *Lactobacillus* strains that do not adhere to HT29 cells do not increase mucin gene expression. Mucin 3 (MUC3) mucin mRNA expression has a direct relationship with extracellular secretion. In coincubation investigations, the same *Lactobacillus* strains that increase MUC3 mucin synthesis inhibited *E. coli* E2348/69 adherence. Probiotics increase MUC3 mucin transcription, translation, extracellular secretion, and epithelial cell adhesion, which improves eukaryotic mucin effects [54], therefore influencing saliva production and their types [55]. After *L. reuteri* treatment, epithelial parotid gland BD-2 expression and levels increase. Studies on epithelial parotid glands have shown a strong connection between elevated BD-2 expression, and reduced *S. mutans*. *L. reuteri* supplementation significantly increases salivary BD-2 levels and glandular BD-2 expression. Probiotics may modify salivary gland epithelial cells such as the parotid gland to increase saliva production [56].

The regular usage of probiotics for *Candida* reduction without side effects is achievable. Probiotics vigorously improve oral health and reduce hyposalivation and dry mouth [55]. Probiotics and xylitol have been shown to reduce *Streptococcus* species in the saliva of orally healthy people. Therefore, probiotics and xylitol may complement each other to stabilize the salivary microbiota [57].

**Table 1:** Alleviative effects of probiotics on the oral cavity, gastrointestinal tract, and liver established in placebo-controlled human trials.

Name of the Probiotic Strains	Age of the Participants	Dose of the Probiotics	Duration of Study	Outcomes of the Study	References
Probiotics Palliate Ailments in the Oral Cavity					
*Lactobacillus.* *reuteri* ATCC55730 and ATCC PTA5289	~38 years	1 × 10^8^ CFU/g each	21 days	Gingival crevicular fluid (GCF) volume increased slightly, with a significantly increase in IL1-β and IL-18 and a significant decrease in IL-8 and MIP1-β also being found.	[58]
*Lactobacillus* *reuteri* PTA5289	31–46 years	1 × 10^8^ CFU/g	14 days	A significant decrease in IL-17, TNF-α, and IL-1β, along with improved clinical indices, including clinical attachment level (CAL), periodontal probing depth (PPD), and sulcus bleeding index (SBI).	[59]
*Lactobacillus* *curvatus* EB10 DSM32307, *Lactobacillus* *rhamnosus* PB01 DSM14869	18–50 years	1 × 10^8^ CFU/g each	28 days	Reduced bleeding on probing (BOP), amount of GCF, and decreased plaque levels.	[60]
*Bifidobacterium* *animalis subsp.* *lactis* DN 173010	16–26 years	1 × 10^8^ CFU/g	28 days	Lowered gingivitis and plaque scores, lessened GCF volume and BOP, and lowered IL-1β concentration.	[24]
*Bifidobacterium lactis* BB-12, *Lactobacillus rhamnosus* GG	13–15 years	4.4 × 10^8^ and 4.8 × 10^8^ CFU/g	28 days	Reduction in the gingival index (GI), plaque and *Porphyromonas gingivalis* in plaque, as well as a reduction in *Aggregatibacter actinomycetemcomitans* and *Fusobacterium nucleatum* in saliva.	[61]
*Lactobacillus acidophilus*, *Enterococcus faecium* *Bifidobacterium infantis*	35–55 years	1 × 10^7^, 1 × 10^6^, and 1 × 10^7^ CFU/capsule	30 days	A significant decrease in BOP after seven days and a reduction in the plaque index (PI), BOP, and periodontal pocket depth (PPD) after 30 days.	[62]
*Bifidobacterium bifidum*, *Lactobacillus Acidophilus*-HS101, *Lactobacillus rhamnosus* GG-HS111	≥60 years	3.3 × 10^7^ CFU/g	60 days	Increased saliva in completely edentulous patients, which can be helpful in hyposalivation/xerostomia patients.	[51]
*Lactobacillus rhamnosus* GG, *Bifidobacterium longum*	3–5 years	7.5 × 10^5^ and 4.5 × 10^5^ CFU/mL of milk	180 days	Significantly decreased *Streptococcus mutans* and pH, as well as the remineralization of 39.4% of caries.	[63]
*Bifidobacterium lactis* Bb-12, *Lactobacillus acidophilus* La-5	6–12 years	1 × 10^6^ CFU/g each	30 days	Reduced *Streptococcus mutans* count after a week and also after 30 days.	[64]
*Lactococcus reuteri* ATCC PTA 5289 and DSM 17938	3–6 years	1 × 10^8^ CFU/g each	28 days	Reduction in Mutans *streptococci* and *lactobacilli* and caries-associated bacterial counts.	[65]
*Lactococcus rhamnosus* GG, *Lactobacillus helveticus*, *Lactococcus lactis*, *Lactococcus rhamnosus* LC705, *Propionibacterium freudenreichii* ssp *shermanii* JS	70–100 years	1 × 10^7^ CFU/g each	120 days	Effectively controlled hyposalivation and oral *Candida* in the elderly.	[55]
*Streptococcus salivarius* M18	6–17 years	1 × 10^9^ CFU/ml	90 days	Increased chances of avoiding the development of new dental caries in kids and reduced risk of tooth decay receptivity.	[66]
*Lactobacillus salivarius* WB21	22–67 years	2.0 × 10^9^ CFU/g	14 days	Significantly decreased organoleptic test scores, the average probing pocket depth, the concentration of volatile sulfur compounds (VSCs), levels of *Fusobacterium nucleatum* and ubiquitous bacteria, which exhibited oral malodor and malodor-related factor control.	[67]
*Lactobacillus salivarius Lactobacillus reuteri*	25–59 years	2 × 10^9^ CFU/g each	90 days	Significantly reduced clinical and microbiological parameters and significantly improved bleeding index (BI), modified gingival index (MGI), and PI, leading to a significant decline in N-benzoyl-DL-arginine-naphthylamide and halitosis.	[68]
*Lactobacillus reuteri* ATCC PTA 5289 and DSM 17938	19–25 years	1 × 10^8^ CFU/g each	28 days	Beneficial for oral malodor and malodourous compounds (other than VSCs) producing bacteria.	[69]
*Streptococcus salivarius* K12	23–44 years	1 × 10^9^ CFU/g	30 days	Significantly decreased immediate organoleptic test (OLT) scores, tongue coating scores, and VSC levels in the absence of tongue coating.	[70]
*Weissella cibaria* CMU	20–39 years	1 × 10^8^ CFU/g	56 days	Significant decrease in OLT and VSC scores, along with bad breath improvement scores being reduced after eight weeks.	[71]
Probiotics Associated with the Small and Large Intestine					
*Bifidobacterium longum* BB536, *Lactobacillus rhamnosus* HN001	37–59 years	4 × 10^8^ and 1 × 10^8^ CFU/g with 1.4 mg vitamin B6	60 days	Reduced abdominal pain, bloating, and disease severity; improved sucralose recovery (colonic permeability); increased relative abundance of hydrocarbons, butanoic, propanoic, and pentanoic acids; and decreased phenol.	[72]
*Lactobacillus* acidophilus subsp. *helveticus* LAFTI L10, *Lactobacillus* acidophilus NCFM	30–60 years	2.5 × 10^9^ CFU/g each	56 days	Significantly decreased flatus and composite scores.	[73]
*Bifidobacterium longum*, *Lactobacillus paracasei*	≥18 years	1 × 10^10^ CFU each	84 days	Reduced symptoms in IBS and addition to the armamentarium of IBS management tools dependent on the IBS subtype.	[73]
*Bifidobacterium bifidum* HI-MIMBb75	≥18 years	1 × 10^9^ CFU/capsule (Non-viable)	56 days	Substantially alleviated IBS and its symptoms and mediated specific beneficial effects independent of cell viability.	[74]
*Bifidobacterium longum*, *Bifidobacterium breve*, *Lactobacillus paracasei* HII01	~60 years	2.0 × 10^10^, 2.0 × 10^10^, and 1.0 × 10^10^ CFU/g	84 days	Improved intestinal barrier function (up to 48%), enhanced short-chain fatty acid levels, improved obesity-related anthropometric biomarkers, and significantly increased high-density lipoprotein–cholesterol.	[75]
*Lactobacillus acidophilus* W37, *Lactococcus lactis* W19 and W58, *Lactobacillus brevis* W63, *Lactobacillus salivarius* W24, *Lactobacillus casei* W56, *Bifidobacterium lactis* W52, *Bifidobacterium bifidum* W23,	18–80 years	2.5 × 10^9^ CFU/g each	180 days	Significantly increased production of reactive oxygen species by neutrophils and serum neopterin levels, maintaining or even improving liver functioning in sturdy cirrhosis with a slight impact on bacterial translocation and gut barrier function.	[76]
*Clostridium butyricum*	≥18 years	1 × 10^6^ CFU/g	15 days	Shortened duration of fever and constipation and significantly decreased bactericides, *Escherichia coli*, and *Enterococcus*.	[77]
*Lactobacillus paracasei* W20, *Lactobacillus plantarum* W1 and W62, *Bifidobacterium bifidum* W23, *Lactobacillus acidophilus* W37 and W55, *Lactobacillus rhamnosus* W71 *Lactobacillus salivarius* W24, *Enterococcus faecium* W54, *Bifidobacterium lactis* W51,	45–65 years	1.1 × 10^9^ CFU/g each	28 days	Increased probiotic strains in stool and improved microbiome composition and functional diversity, successfully modulating the microbiome and ultimately intervening in sepsis.	[78]
*Bifidobacterium* spp.	27–55 years	-	60 days	Decreased plasma levels of hs-CRP, TNF-α, plasma DA0, ET, D-lactic acid, IL-8, and IL-6 and increased CD4/CD8 ratio and CD4+ levels, enhancing the remedying impact in ulcerative colitis patients and regulating T cell frequency, in addition to reducing plasma inflammatory factors.	[79]
*Bifidobacterium longum Enterococcus faecium*, *Lactobacillus acidophilus*, *Lactobacillus plantarum*, *Bifidobacterium lactis*, *Streptococcus thermophilus*,	≥18 years	3 × 10^9^ CFU/g each	56 days	Decreased expression of serum C-reactive protein (CRP), significantly improved endoscopic and clinical activities, and positive impacts on the acute-phase reactants and endoscopic activity levels.	[80]
*Lactobacillus rhamnosus* NCIMB 30174, *Enterococcus faecium* NCIMB 30176, *Lactobacillus acidophilus* NCIMB 30175, *Lactobacillus plantarum* NCIMB 30173,	18–70 years	1 × 10^10^ CFU/g each	28 days	Significantly reduced fecal calprotectin levels in ulcerative colitis patients and decreased intestinal inflammation.	[81]
*Lactobacillus plantarum* 299v	≥18 years	1 × 10^10^ CFU/g	84 days	Reduced enteral nutrition-related gastrointestinal symptoms. Effectively improved the quality of life of cancer patients, nutritional status, and enteral nutrition tolerance.	[82]
Probiotics Tone Liver and Annihilate its Ailments					
*Bifidobacterium* spp., *Lactobacillus* spp., *Enterococcus* spp.	18–59 years	-	90 days	Significantly improved aspartate aminotransferase (AST), NAFLD activity score (NAS), total cholesterol (TC), alanine aminotransferase (ALT), glutamine transferase (GGT), triglyceride (TG) levels, and insulin resistance index (HOMA-IR). Improved liver functions, hepatic fatty deposition, and glucose and lipids metabolism in NAFLD patients, enhancing the therapeutic effects.	[83]
*Pediococcus pentosaceus* CBT SL4, *Lactobacillus paracasei* CBT LPC5, *Lactobacillus rhamnosus* CBT LR5, *Lactobacillus acidophilus* CBT LA1, *Bifidobacterium breve* CBT BR3, *Bifidobacterium lactis* CBT BL3,	19–75 years	1 × 10^9^ CFU/1.4 g each	84 days	Significantly decreased intrahepatic fat fraction after 12 weeks, along with significant triglyceride reduction.	[84]
*Bifidobacterium animalis* subsp. *lactis* BB-12	≥18 years	1 × 10^8^ CFU/g	168 days	Significantly decreased alkaline phosphatase, aspartate aminotransferase, γ-glutamyltransferase, and alanine aminotransferase in serum and reduced NAFLD.	[85]
*Acetobacter* spp., *Bifidobacterium* spp., *Propionibacterium* spp., *Lactobacillus* spp., + *Lactococcu* spp.,	18–65 years	6 × 10^10^, 1 × 10^10^, 3 × 10^10^, 1 × 10^6^ CFU/g	56 days	Significantly reduced the fatty liver index, serum GGT and AST values; diminished chronic systemic inflammatory state; and lowered IL-6 and TNF-α concentrations in NAFLD patients.	[86]
*Lactobacillus lactis* BCMC 12451, *Lactobacillus casei* BCMC 12313, *Lactobacillus acidophilus* BCMC 12130, *Bifidobacterium longum* BCMC 02120, *Bifidobacterium infantis* BCMC 02129, *Bifidobacterium bifidum* BCMC 02290	18 years and above	3 × 10^9^ CFU/g	180 days	Stabilized mucosal immune function, protecting against risen intestinal permeability and playing a complementary role in ministering NAFLD.	[87]
Probiotics as Allayers of Gallbladder and Pancreatic Ailments					
*Clostridium butyricum* MIYAIRI	35.5 ± 9.9	5 × 10^9^ CFU/g	180 days	Decreased incidence of gall bladder disease, adverse drug effects, and poor drug compliance rates, confirming the palliative effects of probiotics.	[88]
*Lactobacillus acidophilus*	48.1 ± 13.8	5 × 10^6^ CFU/g	14 days	Significantly altered serum low-density lipoprotein cholesterol (LDL-C), total cholesterol, total bile acid (TBA), and triglyceride levels. Significantly differed glycoprotein, pH, and free Ca^2+^ of bile. Altered deoxycholic acid, chenodeoxycholic acid, and cholic acid levels, exhibiting the reverse development of bile composition in patients with cholecystolithiasis taking probiotics, thereby diminishing gallstones.	[89]
*Enterococcus faecium Bacillus subtilis*	18–75 years	Manufacturer defined recipe		Significantly reduced length of stay (LOS) and shortened abdominal pain relief and oral feeding duration in patients with acute pancreatitis.	[90]
*Bifidobacterium infantalis*, *Bifidobacterium* *longus*, *Bifidobacterium bifidum*, *Lactobacillus acidophilus*	13–79 years	2.5 × 10^9^ CFU/g	7 days	Significantly reduced immunoglobulins and C-reactive protein expression.	[91]
*Acetobacter* spp., *Lactobacillus* + *Lactococcus* spp., *Propionibacterium* spp., *Bifidobacterium* spp.,	18–75 years	1 × 10^6^, 6 × 10^10^, 3 × 10^10^, 1 × 10^10^ CFU/g	56 days	Significantly improved β-cell function and reduced fasting glucose and hemoglobin A1C levels. Significantly affected chronic systemic inflammation by decreasing pro-inflammatory cytokines.	[92]
*Bacillus mesentericus* TO-A-, *Clostridium butyricum* TO-A, *Lactobacillus* *Sporogenes*, *Streptococcus faecalis* T-110	18–75 years	1 × 10^8^, 4 × 10^6^, 2 × 10^6^, 6 × 10^7^ CFU/g	15 days	Significantly lowered LOS, the duration of antibiotics therapy, and the incidence of postoperative infectious complications in patients with chronic pancreatitis.	[93]
*Lactobacillus casei*, *Bifidobacterium bifidum Lactobacillus acidophilus*, *Lactobacillus rhamnosus*	≥18 years	1 × 10^9^ CFU/g	90 days	Significantly reduced bowel frequency and total cholesterol levels. Significantly increased red blood cells, hematocrit, hemoglobin, albumin, serum magnesium, and total lymphocyte count.	[94]

## 3. Probiotics Proven to Be Beneficial in Small and Large Intestine Disorders

Probiotics have gained attention for their ability to influence indicators of human health. Multiple meta-analyses have exhibited the beneficial effects of probiotics on the symptoms of different gastrointestinal (GI) ailments, including irritable bowel syndrome (IBS) and inflammatory bowel disease (IBD). Further meta-analyses have been conducted to evaluate the effectiveness of probiotic variations depending on the specific strain and the disease being targeted, with increasing compelling evidence portraying probiotics’ strain-specific effects in alleviating symptoms related to specific conditions or ailments (Table 1) [15,95,96,97].

The duodenum, jejunum, and ileum of the small intestine (SI) process and absorb macro- and micronutrients. The SI components’ luminal environments vary, affecting microbial abundance in each segment. Ileum bacteria concentrations rise to 10^8^ bacteria/mL from 10^3^ to 10^4^ in the duodenum and jejunum. GI tract bacteria density grows along its length but stays low compared to colon bacteria concentration, which is 10^11^ bacteria per milliliter.

The GI tract mucosal epithelium protects the host from the environment. The intestinal barrier consists of junctional complexes (including adherens junctions, desmosomes, and tight junctions (TJs)), antimicrobial peptides (AMPs), the mucus layer, and the commensal gut microbiota. These adaptable parts maintain barrier haleness [98]. Damage to the epithelial mucosa or changes in dysbiosis, nutrition, or inflammation may increase barrier permeability [96].

### 3.1. Lactobacillus spp. as Small Intestinal Alleviators

**Figure 4:**
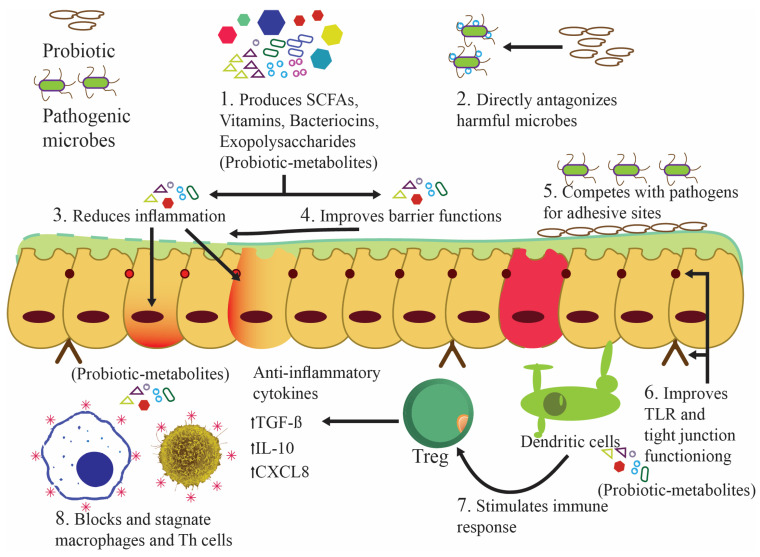
The palliative effects of probiotics on intestinal integrity and the working of the anti-inflammatory mechanism in the gastrointestinal tract.

The administration of three probiotic strains (*L. reuteri* G8-5, G22-2, and *Lactobacillus salivarius* G1-1), in comparison to an antibiotic control group, assisted in the expression of several pathogen defenses, the maintenance of cell structure integrity, and the maintenance of protein cell stability (Figure 4) [99]. Administrating *Lactobacillus rhamnosus* GG before challenging pigs with *Salmonella infantis* decreased *S. Infantis*-induced IL-7Rα production in the jejunum and T cells+ interferon-gamma (IFNγ)+ clusters of differentiation 4 (CD4) in Peyer’s patches. These facts establish the immunological benefits of *L. rhamnosus* GG as a probiotic and the complexity of its interactions [100]. Therefore, probiotics, especially LAB, protect the small intestine by increasing microbial diversity, homeostasis-related protein expression, and immune system integrity (Table 1).

### 3.2. The Palliative Intestinal Permeability of Lactobacillus spp.

The selective permeability of the intestinal barrier lets water and nutrients flow while blocking germs and poisons. Tight junctions (TJ) mainly control paracellular permeability. By causing inflammation and limiting nutritional availability, prolonged gut barrier disruption may cause GI and autoimmune illnesses. Probiotics maintain intestinal barrier health throughout the intestines. If the microbiome is balanced, probiotics boost butyrate production, strengthen TJ proteins, and protect the mucosal lining. Thus, probiotics improve nutrient absorption [101].

A recent study examined *L. reuteri* LR1’s impact on the characteristics of the small intestine, particularly intestinal permeability. In this study, the weaned pigs, who received food fortified with *L. reuteri* LR1, exhibited higher mucosal TJ protein expression and villus height/crypt depth ratios in the jejunum and ileum than those fed antibiotics [102]. In another study, mice given lipopolysaccharide to hasten barrier dysfunction, followed by *L. rhamnosus* GG and *L. reuteri* ZJ617 as mitigators, showed unique attributes. Claudin-3 and occludin decreased after lipopolysaccharide consumption, and their functions were restored after intervention with probiotics. Therefore, the administrated probiotic strains effectively mitigated this dysfunction [103]. In another study, heavy kanamycin dosages compromised mice’s intestinal barriers. The LAB-fed mice exhibited higher ileal occludin and zonulin-1 expression than the control-fed mice. Participants’ Peyer’s patch cells had more elevated blood immunoglobulin A levels, showing that LAB mitigates kanamycin’s devastating effects [104]. Human research confirms animal studies’ claim that LAB can maintain barrier integrity (Figure 4). As a probiotic, *Saccharomyces boulardii* CNCM I-745 prevents and treats diarrhea caused by antibiotics, infections, and functional factors. *S. boulardii* CNCM I-745 improves intestinal microbiota and epithelial barrier abnormalities in diverse illnesses. The probiotic yeast *S. boulardii* CNCM I-745 helps maintain or repair the intestinal barrier in various ailments [105].

### 3.3. Probiotics as Lenitives against Impaired Nutrient Absorption and Chronic Diarrhea

Small intestinal bacterial overgrowth (SIBO) has been identified as a potential etiology for impaired nutrient absorption and chronic diarrhea. The quantitative characteristics of small intestinal bacterial cultures do not affect the functional gastrointestinal symptoms of SIBO. These symptoms do correlate with a microbial imbalance in the small intestine. Different levels of nutrient malabsorption cause weight loss and vitamin-deficient neuropathies [106].

**Figure 5:**
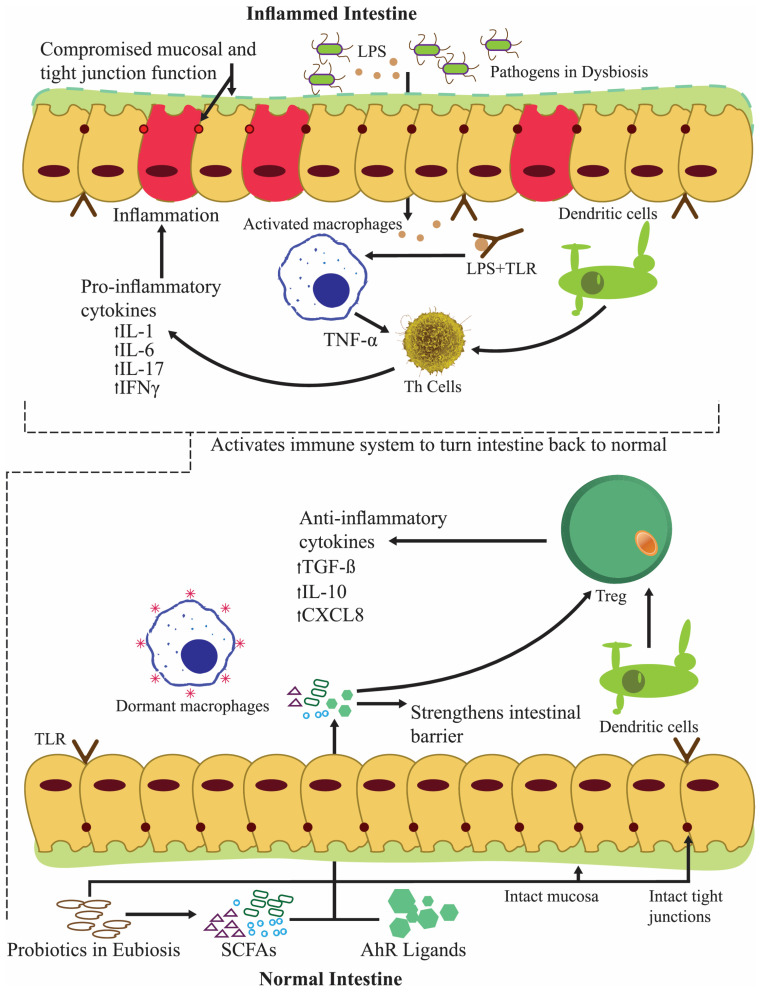
Anti-inflammatory activity of probiotics during inflammatory bowel syndrome and reverting the intestine to normal functioning.

Individuals with SIBO typically exhibit luminal content bacterial concentrations ranging from 10^5^ to 10^6^ bacteria per milliliter, which is approximately 2 to 3 log10/mL higher than those observed in healthy individuals. The bacteria found in the small intestine of patients with SIBO are typically the same as those found in the oropharynx and colon. Rifaximin is the most common SIBO treatment. However, it can disrupt good bacterial populations and induce antibiotic-associated diarrhea and *Clostridium difficile* infections. Thus, probiotics are being evaluated to treat bacterial giantism and restore small intestine commensal microorganisms (Figure 4 and Figure 5) [107].

The complexity of irritable bowel syndrome (IBS) is compounded by various etiologies and symptomatic subtypes. Altered bowel habits, including diarrhea, constipation, or both, as well as stomach pain, characterize IBS [108]. SIBO can coexist with IBS. The evidence for small intestine dysbiosis in IBS is strong. However, a lack of knowledge about probiotic strains, dosages, and therapy duration hinders the potential of probiotic treatment for IBS [109]. Treatment with *Bacillus* spp. spores has been shown to improve IBS patient’s quality of life, likely due to alterations in the gut microbiota (Table 1) [110].

### 3.4. Probiotics Modulating Large Intestinal Microflora

Oral probiotic bacteria support and modify the metabolic processes and composition of the microflora of the large intestine. Fermentation by large intestine microorganisms aids digestion. Lowering the intestinal pH makes it more acidic, making it unsuitable for dangerous species. The microflora also guards against pathogenic microorganisms, preventing illnesses. In addition, they actively mature immune system components. Lactic acid bacteria play a crucial role in the gut microbiota, influencing the landscape for health advantages. Regular probiotic bacteria consumption maintains their health advantages.

### 3.5. The Alleviating Influence of Lactobacillus spp. on Colitis

In specific pathogen-free environments, mice lacking the interleukin (IL)-10 gene (IL-10-/-) develop colitis, while in sterile environments, they do not. *Lactobacillus plantarum* reduced colonic inflammation in SPF IL-10-/- mice by lowering mucosal IFN-gamma, immunoglobulin G2a, and IL-12 levels. Monoassociation with *L. plantarum* in gnotobiotic IL-10-/- mice causes considerable immune system activation but minimal colitis. In one specific study, probiotics administration in germ-free mice significantly lowered histologic colitis scores. These facts signify that *L. plantarum* reduces immune-mediated colitis and clinically treats inflammatory bowel diseases (Table 1) [111].

Another trial investigated whether exogenous *Lactobacillus* could help rats with acetic acid-induced colitis. Four days after acetic acid administration, uniform colitis, a three-fold increase in colonic tissue myeloperoxidase (MPO) activity (an indicator of neutrophil infiltration), and a six-fold increase in plasma exudation occurred. *L. reuteri* R2LC intracolonic injections after the administration of acetic acid alleviated colitis. Thus, *Lactobacillus* nearly normalized mucosal permeability, MPO activity, and morphologic score. The soothing impact of exogenous *L. reuteri* R2LC in preventing acetic acid-induced colitis in rats is evident [112].

Certain probiotics modulate allergic inflammation, reducing inflammation outside the gut (Table 1). The aggregate effects of these probiotic strains help neonates adjust during weaning, which begins with antigen sensitivity. Probiotics could help develop new allergy-fighting foods [113].

### 3.6. Bifidobacterium, Lactobacillus, and Other Probiotic spp. Modulating Gastrointestinal Cancers

Many gastrointestinal (GI) malignancies exist, including spontaneous and hereditary variants. Cancer can develop when genetic and environmental factors turn healthy tissue into a precursor or premalignant condition. Specific tissue and cell types have partially known genetic pathways of GI malignancies of various sources, and they share some similarities [114]. Probiotics are utilized as supplements, in line with the progress made to develop new diagnostic and therapeutic methods for GI cancers.

Different researchers have examined how probiotics help reduce symptoms and improve quality of life in colorectal cancer patients at various stages. According to one study, Lacidofil supplements reduced gastrointestinal discomfort and improved functional well-being in colorectal cancer patients [115]. Elevated serum levels of zonulin, a haptoglobin-2 precursor, have been linked to the presence of gastrointestinal cancers, inflammatory diseases, and autoimmunity [116]. Zonulin levels dropped significantly in colorectal cancer patients receiving *B. longum*-88, *L. acidophilus*-11, and *L. plantarum*. In addition, probiotics alleviate infection problems, decrease antibiotic use, and alleviate postoperative fever. Probiotics also inhibit the p38 mitogen-activated pathway, which controls cell differentiation, inflammation, growth, and death [117]. The use of *E. faecalis*, *L. acidophilus*, and *B. longum* shorten the time until first bowel movement, gas, and diarrhea (Yang et al., 2016). *L. rhamnosus* GG supplementation has been shown to significantly reduce diarrheal episodes in colorectal cancer patients using 5-Fluorouracil, a chemotherapy medication known to cause diarrhea [118]. Offering colorectal cancer patients a probiotic mixture of *Bacillus mesentericus* TO-A, *Clostridium butyricum* TO-A, and *Enterococcus faecalis* T110 reduced superficial incisional infection rates [119]. A combination of *E. faecalis*, *L. acidophilus*, and *B. longum* has been shown to change the gut flora of colorectal cancer patients. Moreover, those probiotics also reduce *Fusobacterium*, a cancer inducer, taxon secretion [120]. In colorectal cancer patients, a mixture of probiotics (*L. plantarum*, *L. acidophilus*, *S. boulardii*, and *B. lactis*) reduce pneumonia, mechanical ventilation, surgical site infections, and anastomosis leakage [121].

## 4. Probiotics Tone Liver and Annihilate Its Ailments

As the leading cause of chronic liver diseases, non-alcoholic fatty liver disease (NAFLD) is a global public health issue. The term “hepatic conditions” covers many liver-related issues. These conditions range from simple steatosis, which is the deposition of lipids on more than 5% of the liver without other causes, to severe forms like non-alcoholic steatohepatitis (NASH), hepatocellular carcinoma (HCC), cirrhosis, and fibrosis.

### Probiotics Rectifying Non-Alcoholic Fatty Liver Disease (NAFLD) and Alcoholic Liver Disease (ALD)

Gut bacteria play a significant role in developing and progressing liver disease in metabolic syndrome and NAFLD, which affect children and adults. NAFLD risk increases due to dysbiosis, intestinal barrier dysregulation, and gut bacterial overgrowth. According to clinical guidelines, lifestyle changes and diet are the primary treatment avenues for NAFLD and related disorders. Patient non-adherence hinders these therapies’ efficacy, generating poor outcomes. To tailor NAFLD treatment, it is necessary to investigate various other therapies.

**Figure 6:**
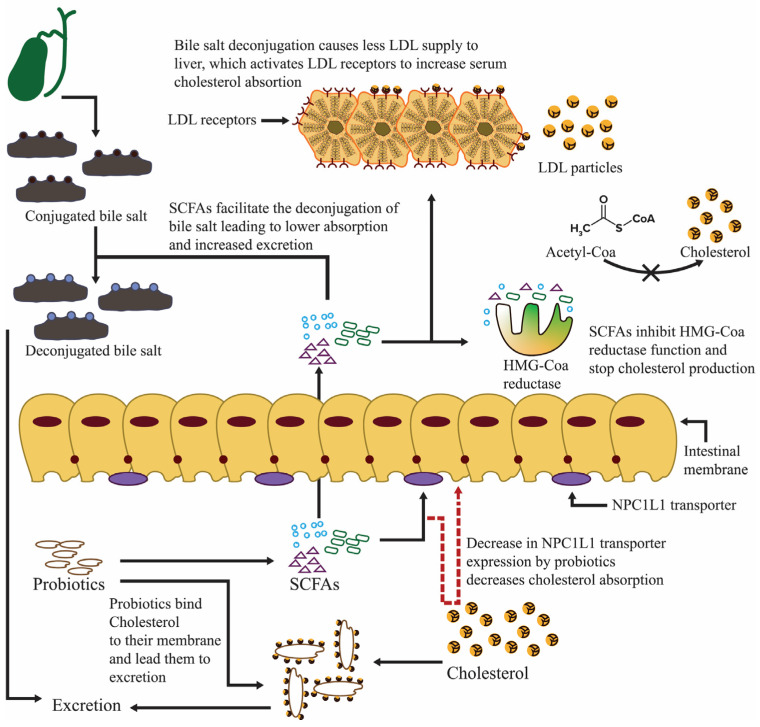
The preventive mechanism of probiotics, which hinders NAFLD prevalence.

Numerous clinical research studies support probiotic supplements as a safe and effective treatment method. These findings highlight the untapped potential of restoring intestinal microbiota as a standard therapeutic therapy for NAFLD. Additionally, probiotics can be used alone or in combination with NAFLD treatments (Figure 6) [122].

In one study, probiotic postadministration multivariate analysis demonstrated significantly lower alanine aminotransferase and antipeptidoglycan-polysaccharide antibodies despite changes in visceral fat and body mass index (BMI) z score. US light liver readings and TNF-α remained stable. *L. rhamnosus* GG, a potent probiotic, should be considered to treat hypertransaminasemia in hepatopathic obese youngsters with rebellious lifestyles [123].

Probiotics significantly decrease liver enzymes in non-alcoholic steatohepatitis, particularly alanine aminotransferase (ALT), and increase the expression of aspartate aminotransferase (AST). Dyspepsia symptoms also improve. The efficacy, safety, tolerability, affordability, long-term appropriateness, and potential multilevel downregulation of inflammatory mediators make probiotics a promising treatment [124].

Multistrain probiotics work better. Multistrain probiotics, *L. rhamnosus* DSMZ 21,690, *L. acidophilus* ATCC B3208, *Bifidobacterium lactis* DSMZ 32,269, and *B. bifidum* ATCC SD6576, intervention can reduce ALT levels, intrahepatic fat content, and sonographic lipid profiles. A “Symbiter” containing 14 live probiotic strains of *Acetobacter*, *Propionibacterium*, *Bifidobacterium*, and *Lactobacillus* + *Lactococcus* improved tumor necrosis factor (TNF)-α, IL6, aminotransferase activity, and hepatic steatosis in NAFLD patients [86]. Lepicol probiotics lower liver triglycerides and AST levels in NASH patients, as confirmed by histology [125]. *Streptococcus*, *Bifidobacterium*, and *Lactobacillus* probiotics have been shown to improve hepatic fat content, aminotransferase levels, total cholesterol, and a homeostatic model’s assessment of insulin resistance [126]. Probiotics improve insulin sensitivity and reduce TNF-α levels in NAFLD patients (Table 1, Figure 6). However, probiotics only improve dyslipidemia in Spanish and Italian people, suggesting that ethnicity has a connection with low-density lipoproteins (LDL), high-density lipoproteins (HDL), and triglyceride levels [127].

The most thoroughly studied probiotic with many strains, VSL#3, can protect the intestinal barrier. Furthermore, it reduces oxidative/nitrosative stress and endotoxemia, improving liver health in chronic liver disease patients [128] VSL#3’s potential to modify the gut microbiota with *B. longum* is intriguing because it produces conjugated linoleic acid, which changes liver fatty acid composition. These facts support the idea that gut–liver interaction is essential in designing NAFLD treatments [122,129]. The co-administration of *B. longum* and fructooligosaccharides (FOS) improved metabolic, inflammatory, and fibrosis scores in NASH patients [130].

## 5. Probiotics as Allayers of Gallbladder Ailments

The prevailing consensus in the scientific community is that dietary habits, particularly ingesting a high-fat diet over an extended period, constitute a substantial risk factor for developing cholesterol gallstones [131,132].

Probiotics significantly lower gall bladder disease prevalence when compared with digestive enzymes. This notion implies that probiotics are not inferior to other therapies. Probiotics also improve drug compliance compared to other treatments and lower pharmaceutical side effects. Therefore, probiotics are pretty effective in preventing gallbladder diseases (Table 1) [88].

### 5.1. Probiotics Repress Bile Acid Production and Diminish Gallstones

Probiotics prevent gallstones by lowering cholesterol. Additionally, probiotics may alter the profiles of serum bile acids by decreasing the proportion of deoxycholic acid in serum [133]. Oral *Clostridium butyricum* Miyairi therapy systematically reduces gallstone cholesterol content, incidence, and index in mice with cholesterol cholelithiasis. Probiotic treatments also dissolve gallstones well [134]. The intestinal prevalence of *C. butyricum* Miyairi No. 588 increases bile acid excretion and inhibits gallstone formation in mice [135].

Certain gut microorganisms produce cholesterol reductase, which converts cholesterol into insoluble coprostanol. The fecal excretion of coprostanol lowers exogenous cholesterol [136]. Probiotics, specifically *L. acidophilus*, *B. lactis*, VSL #3, and *L. plantarum*, noticeably reduce serum cholesterol levels [137]. Probiotics prevent and treat lipid-related diseases without medication. A BSH-positive *Lactobacillus* strain extensively diminished the cholesterol levels of hypercholesterolemia patients [138]. Farnesoid X receptor (FXR) agonists may reduce gallstones by adjusting bile salts and phospholipids [139]. Chenodeoxycholic acid (CDCA) and cholic acid (CA) help activate FXR. The gut bacteria metabolize these bile acids to produce secondary ones. Thus, metabolic processes affect FXR activity and signaling [140]. Probiotics and dietary changes modify the ‘gut microbiota–bile acid–host’ signaling connections. These treatments provide unique ways to treat bile acid metabolism problems [141].

It has been shown that *L. acidophilus* ATCC 43121 diet supplementation lowers blood low-density and total lipoprotein cholesterol by lowering 3-hydroxy-3-methylglutaryl coenzyme expression in Mice fed with a high-cholesterol and high-fat diet. Moreover, *L. acidophilus* ATCC 43121, along with *L. fermentum* MF27, lowers cholesterol and decreases the expression of gel-forming mucins like MUC5B and MUC5AB. Thus, the consistent dosing of these probiotics inhibits cholesterol gallstones. Both probiotic species also improve serum biochemical indices without affecting growth. Reduced liver HMG CoA R expression causes the serum to lower cholesterol, especially after ingesting *L. acidophilus* ATCC 43121. These traits may also reduce gallbladder gel-forming mucins like MUC5B and MUC5AC. Thus, consuming lactobacilli regularly helps prevent cholesterol gallstones in therapeutic circumstances (Table 1) [142].

### 5.2. Probiotics’ Connection with Bacterial Translocation and Acute and Chronic Pancreatitis

Acute pancreatitis (AP), a common gastrointestinal illness caused by gallstones and alcohol intake, can lead to hospitalization [143]. AP begins with acinar cells converting pancreatic enzymes from inactive to active, causing pancreatic tissue to autodigest. The release of proinflammatory cytokines such as IL-1, IL-6, IL-8, and TNF-α causes pancreatic inflammation [144,145].

Acute pancreatitis (AP) damages the pancreas. Gram-negative bacterial infections activate digestive enzymes pathologically, causing inflammation and cell signaling alterations. This increases intestinal permeability and lets microbes, endotoxins, and antigens into the pancreas, causing BT and acute illness. Direct transmural migration into the retroperitoneum or peritoneal cavity might lead to pancreatic, hematogenous, or lymphatic dispersion. It causes gut barrier breach, small bowel hypomotility, and systemic immunosuppression. Stellate cell and fibrotic tissue activation from recurrent pancreatitis induce chronic pancreatitis (CP). These findings suggest that acute, recurrent, and CP pancreatic cancer can metastasize [146,147,148].

Probiotics protect healthy gut ecology. The disruption of gut bacterial microflora homeostasis may increase bacterial translocation by altering barrier function. Bacterial translocation (BT) increases inflammation, leading to CP and pancreatic cancer [149,150]. Giving acute pancreatitis patients *L. plantarum* 299 reduced pancreatic sepsis and surgical procedures in [151]. Treating severe acute pancreatitis with synbiotics, including prebiotic fibers and LABs, reduces mortality rates. The probiotics *S. boulardii* and ciprofloxacin have been shown to reduce acute necrotizing pancreatitis histopathology scores. Moreover, the enteral feeding of probiotics is more beneficial than parenteral feeding. This method of probiotics feeding reduces the severity of pancreatic conditions like inflammation, edema, fibrosis, parenchymal necrosis, acinar cell loss, ductal damage, PMNL, MNL, vacuolization, and atypical reactive regeneration. These enteral administrations also prevent pancreatic cancer [152].

Probiotics decrease duodenal bacterial overgrowth and pancreatic translocation. Health scores and late-phase mortality have been shown to improve significantly. In acute pancreatitis, altering intestinal microbiota using probiotic species reduces BT, morbidity, and mortality [150].

The collapse of the intestinal barrier causes BT to enter into the circulation and necrotic tissues from the digestive tract, leading to pancreatic tissue infection. Typically, the pancreas has no well-defined microbiome. However, gastrointestinal tract dysbiosis commonly affects it. Pancreatic macrophages release TNF, IL-6, and IL-1 in necrotizing pancreatic tissues when bacterial antigens and endotoxins enter the portal circulation. These cytokines contribute to chronic pancreatitis and pancreatic tumors. Due to the lack of a screening modality and the poor outcomes of pancreatic cancer therapies, effective primary prevention techniques such as probiotic interventions are the best way to reduce morbidity and mortality [148].

In animals suffering from severe pancreatitis, *Pseudomonas*, *Enterococcus faecalis*, *E. coli*, and *Proteus* predominate. Animals with *L. plantarum* 299v ‘umbrella’ reduce mesenteric lymph node cultures. In animal pancreatic tissue cultures with *E. faecalis* or *Escherichia coli*, *L. plantarum* 299v effectively reduces microbiota translocation. Based on these facts, probiotic bacteria may replace antibiotics as a therapeutic strategy [153].

Probiotics significantly reduce pancreatic and oxidative damage. Probiotics strongly block AP-induced NF-kappaB activation, reduce AP-induced lipid peroxidation and glutathione depletion, and increase glutathione levels. Probiotics increase glutathione production, which may reduce inflammation and acinar cell injury. These actions likely mitigate oxidative stress and improve acute pancreatitis [154].

### 5.3. Probiotics Lower the Risks of Organ Failure and Systemic Inflammatory Response Syndrome

The administration of synbiotics has been shown to lower mortality, septic complications, and multiorgan failure (MOF) in pancreatic patients. Synbiotic treatment reduces systemic inflammatory response syndrome and MOF rates. Moreover, the early nasojejunal feeding of synbiotics can lead to an avoidance of organ failure in severe acute pancreatitis. Pancreatic necrosis infection may also influence early-stage organ failure [151].

Probiotics significantly reduced total leucocyte and neutrophil counts in patients of the same demographic and the same severity of pancreatitis. Hospitalization length (LOH) also shortened, significantly reducing non-septic morbidity and intensive care unit (ICU) stays. Synbiotics have also been shown to reduce septic complications in moderately severe and severe acute pancreatitis patients. Synbiotics significantly reduced LOH without reducing fatality rates or medical interventions (Table 1) in [155].

Pre/pro/synbiotics reduce hospital stays significantly, which proves their efficacy. Pre/pro/synbiotics also lower severe acute pancreatitis (SAP) patients’ risk of MOF and LOH. Pre/pro/synbiotics do not worsen SAP patients’ clinical outcomes. These individuals have a lower organ failure risk and shorter LOH [156].

High-temperature heating transforms heterocyclic aromatic amines (HCAs) in beef [157,158] into active derivatives, including pyrolyzates such as 3-amino-1-methyl-5H-pyrido-[4,3-b]indole [Trp-P-2], 3-amino-1,4-dimethyl-5H-pyrido-[4,3-b]indole [Trp-P-1] and compounds that promote tumorigenic mutations [159]. Commensal bacteria, especially LAB, retain or catabolize these mutagenic chemicals [160]. Probiotics bind or degrade HCAs, which then eliminate carcinogens from the body. Probiotics, especially LAB, adhere to carcinogenic HCAs formed during protein-rich food heating [161]. Zhang et al. observed a decrease in the genotoxicity of Trp-P-1, a nitroso compound, following its interaction with *L. acidophilus* and *Bifidobacterium* species [162]. The antimutagenic elements from *L. plantarum* KLAB2 affect N-methyl-N’-nitro-N-nitroso-guanidine’s mutagenesis effects in *Salmonella enterica* strain TA100 cells. The anti-mutagenic property is attributed to three glycoproteins located outside the bacterial cell wall [163].

Interestingly, probiotics reduce the perniciousness of toxic heavy metals and fungal mycotoxins, which contribute to pancreatic carcinogenesis [164]. Additionally, the influence of *Propionibacteria*, widely recognized dairy probiotics, in reducing cyanotoxins such as microcystin-LR, lead, and cadmium is well established [165,166]. Reducing carcinogenic chemical bioavailability reduces pancreatic cancer risk. *L. rhamnosus* GG’s cellular degradation of aflatoxin B1 is evident. *L. acidophilus* 24 and *S. cerevisiae* CECT 1891 remove fumonisin from the cells [167,168].

## 6. Probiotics Fortify the Respiratory Tract and Alleviate Rhinosinusitis and Rhinitis

Inflammatory illnesses affecting the upper respiratory tract (URT), including chronic rhinosinusitis (CRS), acute rhinosinusitis (ARS), and rhinitis, have a substantial impact on public wellness and significantly contribute to healthcare expenditures. Rhinitis, a URT disorder, is characterized by the symptomatic inflammation of the nasal lining caused by infectious agents, allergens, hormones, and medicines [169]. Allergic rhinitis (AR), a non-infectious form of rhinitis, is also common. Rhinosinusitis causes paranasal sinus and nose swelling. Experiencing more than two symptoms, including nasal blockage or drainage, is an indicator of rhinosinusitis. Illnesses lasting more than 12 weeks become CRS [170].

The indigenous microflora in the URT of children and healthy adults includes LAB members such as *Lactococcus*, *Dolosigranulum*, and *Lacticaseibacillus* species. *Lactobacillus* species in the nasopharynx and tonsillar crypts of adults and children from China, Canada, and Belgium have been found [171,172,173,174,175].

A decrease in specific LAB taxa such as *Latilactobacillus sakei* in CRS patients suggests sinus health benefits. In a study involving the use of a mouse model used to investigate sinus infection, the findings showed that *L. sakei* ATCC15521 protects sinus mucosa from *C. tuberculostearicum* pathogenesis after nasal inoculation [176].

Various LAB taxa are more common in the URT than Lactobacillaceae. *Dolosigranulum pigrum*, a neglected species, is being considered for the establishment of the URT’s next-generation probiotic. The main reason for this is its abundance, which can reach 50% in people with normal URTs. It is often more common in healthy people than sick people, suggesting a link to URT health [172,177,178,179].

### 6.1. Probiotics Have Yielded Encouraging Findings against Asthma

Asthma and other lower airway comorbidities must be considered when assessing the therapeutic effects of probiotics in chronic inflammatory diseases of the URT, such as CRS and allergic rhinitis [169]. The initial clinical trials have exhibited that *Lactobacillus gasseri* PM-A0005 has therapeutic effects in asthmatic children. *L. gasseri* PM-A0005 improves asthma, airway function, and, particularly, peak expiratory flow rates. Additionally, the probiotic strain significantly decreases the expression of pro-inflammatory cytokines such as IL-13, IL-12, IFN-γ, and TNF-α (Table 2) [180]. An oral mixture of *B. bifidum*, *L. delbruecki* ssp. *bulgaricus*, and *L. acidophilus* has been shown to improve pulmonary function and reduce asthma exacerbations [181]. Synbiotic therapy affects asthmatics and dust mite allergy sufferers. Fructo- and galactooligosaccharide and *Bifidobacterium breve* M-16V significantly improve serum IL-5 and initial lung function (Table 2) [182].

### 6.2. Probiotics Counter Allergy Illnesses

High rates of allergic sickness are becoming a global health issue, especially in highly developed places like North America, Western Europe, and Australasia, where over 40% of the population may feel its effects [228]. Due to industrialization and Westernization, international patterns imply that environmental changes affect immune function regardless of genetics. However, growing evidence suggests that non-Caucasians may be more susceptible to allergic illnesses. This discovery is concerning, especially in densely populated, rapidly urbanizing locations [229,230].

*Lactobacillus* F19 shows a higher IFN-gamma/IL4 mRNA, implying that probiotics prevent early allergy diseases like eczema during weaning. This probiotic elevates the Type 2 T helper (Th2)–Type 1 T helper (Th1) ratio, suggesting that *Lactobacillus* F19 improves T cell-mediated immunity [231].

*L. plantarum* NumRes8 and *B. breve* M-16V inhibit several parameters, including methacholine responsiveness, bronchoalveolar lavage fluid eosinophil activity, and ovalbumin (OVA)-specific immunoglobulin E (IgE) and immunoglobulin G1 (IgG1) levels. B. *breve* M-16V also reduces IL 4, 5, or 10 and acute allergic skin reactions associated with OVA-induced asthma. Overall, *B. breve* M-16V is the most potent antiallergic strain [232].

*B. breve*-12 and *L. rhamnosus* GG reduce asthmatic symptoms such as pulmonary eosinophilia, antigen-specific immunoglobulin E production, and airway reactivity. Mesenteric lymph node cells produce fewer Th-2 cytokines (IL-4, IL-5, and IL-10), and spleen cells proliferate less in response to antigen-specific recall. The oral treatment of *L. rhamnosus* GG reduces allergen-induced proliferation. The mesenteric lymph nodes’ CD4+/CD3+ T cells, which release transforming growth factor-beta, increase with this suppression. Probiotics reduced allergic sensitization and airway disease in a mouse model of asthma. Promoting T regulatory cells, which increase TGF-beta production, achieves this effect [233].

Probiotics probably affect Th17, a niche subgroup of CD4+ T lymphocytes linked to allergic problems. Oral *Enterococcus faecalis* FK-23 (LFK) relieves inflammatory cell accumulation, bronchoalveolar lavage fluid (BALF), and airway resistance in lung tissue. In mice challenged with OVA, LFK also lowered the percentage of CD4+ cells expressing IL-17 in the lungs, spleen, and stomach. Oral leukotriene receptor antagonists reduce asthma symptoms and Th17 cell proliferation [234].

## 7. Probiotics Combat Osteoporosis and Build Up the Skeleton

The number of fractures resulting from osteoporosis has exceeded 2 million annually, and therapeutic approaches for osteoporosis prevention and treatment are manifold. Early intervention involves asking people to exercise, quit smoking, and take vitamin D and calcium supplements [235]. Patients with a high fracture risk are treated with medications and biologics. These treatments inhibit bone resorption or encourage development [236]. Given the momentous prevalence of bone decreases, it is crucial to identify additional osteoporosis treatment methods and targets [237].

*L. paracasei* DSM13434 or a mix of *L. plantarum* DSM 15313, DSM 15312, and *L. paracasei* DSM13434 (*L*. mix) have been shown to protect mice from bone resorption and OVX-induced cortical bone loss. *L*. mix and *L. paracasei* DSM13434 have been shown to elevate cortical bone mineral content in OVX mice. *L*. mix and *L. paracasei* DSM13434 lower the urine fractional excretion of calcium and resorption marker C-terminal telopeptides serum levels. Probiotics reduce IL-1β and TNFα levels and increase OPG expression. Probiotic treatment upholded regulatory T cell frequency in VEH-treated mice’s bone marrow. Overall, *L*. mix and *L. paracasei* DSM13434 reduce cortical bone loss, alter bone’s immune system, and reduce bone resorption in mice [238].

Fucooligosaccharides in yacon flour make it a prebiotic suitable for synbiotic food production. *B. longum*, along with yacon flour or diet, elevates tibia Mg, Ca, and P and enhances bone strength. Yacon flour assists in developing heavier anaerobes and cecums. Using *B. longum* in yacon flour or diet increases cecal propionate levels. Yacon flour and *B. longum* increase bone mineral levels, which helps prevent osteoporosis [239].

Insufficient estrogen accelerates osteoporosis, which causes bone resorption and inflammation. *L. reuteri* ATCC PTA 6475 releases immunomodulatory substances and protects mice from bone loss. *L. reuteri* reduces the receptor activator of nuclear factor kappa beta, Tartrate resistant acid phosphatase 5, and osteoclastogenesis. *L. reuteri* ATCC PTA 6475 inhibits OVX-induced bone marrow CD4+ T-lymphocytes, which increase osteoclastogenesis. *L. reuteri* ATCC PTA 6475 also suppresses osteoclastogenesis in vitro, affects the stomach microbial populations, and decreases bone resorption and loss in estrogen-deficient patients. *L. reuteri* ATCC PTA 6475 is a cost-effective way to reduce bone loss in postmenopausal women [240]. *L. plantarum* (NTU 102)-fermented soy milk or *L. paracasei* (NTU 101) increase bone trabecular number and volume fraction (BV/TV) [241].

### 7.1. Probiotics Meliorate Bone Health

Probiotics also contribute to improving bone health. *L. reuteri*, a probiotic recognized for its anti-inflammatory and bone health properties, protects mice from type 1 diabetes-induced bone loss and marrow obesity. *L. reuteri* prevents Wnt10b downregulation in type 1 diabetic bone because a lower bone-specific Wnt10b expression is linked to osteoporosis. *L. reuteri* substantially reduces the negative impact of TNF-α on Wnt10b expression and osteoblast formation. Probiotics protect bones from type 1 diabetes-induced degradation and elevate bone health [242].

Orally administrated *L. reuteri* ATCC 6475 elevates bone mineral density, content, number, thickness, and femoral and vertebral trabecular bone density [243]. During the onset of mild inflammation in mice, the administration of oral *L. reuteri* was shown to lead to improved bone health. Moreover, female mice ingesting probiotics with mild inflammation from a dorsal surgical incision (DSI) have higher bone density. Probiotic administration in DSI mice elevates femoral trabecular bone density, mineral apposition rates, and trabecular numbers [244]. Probiotic administration with no prior health issues yields a higher anti-inflammatory response. However, *L. reuteri* diminishes inflammation and improves bone production with enhanced inflammation in females with intact estrogen levels. Thus, *L. reuteri* improves bone density.

*L. gasseri*, *L. reuteri*, and *L. casei*-enriched yogurt increase calcium absorption in mice. This intervention increases bone mineral content (BMC), while *L. rhamnosus* (HN001) improves magnesium and calcium retention [245]. *B. longum* and other *Lactobacillus* strains also improve bone health. *B. longum* (ATCC 15707) increases tibia phosphorus, magnesium, and calcium levels. Probiotic supplementation also enhances bone strength [239]. Mice ingesting *B. longum*-fermented broccoli feeding on a cholesterol-rich diet demonstrated reduced tartrate-resistant acid phosphatase-positive osteoclasts in [246].

Lactobacilli reduces the adverse effects of estrogen deprivation on trabecular bone density. *L. rhamnosus* GG and VSL#3 administration positively impact femoral trabecular thickness, bone density, and number reduction. Genetic changes in probiotics cause a loss in their probiotic ability, such as *L. rhamnosus* GG pili mutant (ΔSpaC) and *E. coli* DH5alpha, which did not reduce bone loss in one specific study. The ingestion of VSL#3 and *L. rhamnosus* GG lowered blood collagen type I C-telopeptide levels, which indicates osteoclast-mediated bone resorption [247]. This implies that probiotics exert their impact on bone loss by reducing osteoclast activity.

*B. longum* affects bone mineral content, bone structure, bone density, bone remodeling, and the expression of genes associated with osteoclasts and osteoblasts by increasing bone density, trabecular number, and thickness. *B. longum* supplementation also increases femoral strength. *B. longum* therapy reduces serum C-terminal telopeptide and attenuates a decrease in osteoblast surface and an increase in osteoclast surface relative to the femur bone. These notions suggest that probiotics influence osteoclast activity and growth [248].

### 7.2. Probiotics Palliate Rheumatoid Arthritis

Rheumatoid arthritis (RA) is a persistent disorder distinguished by inflammation, neuropathic uncomfortableness, rigidity, fractures, diminished functionality, and cartilage degeneration, which collectively lead to impaired physiological performance [249,250]. RA affects 1% of those aged 20–40 worldwide; however, it is more common in those 75 and older [251,252]. Psychological problems, asthma, cancer, heart illnesses, hypertension, diabetes, nephritis, and lung cancer/COPD are common comorbidities in RA patients. Comorbidities increase RA patients’ risk of death [253].

RA patients have higher amounts of *Shigella*, *Escherichia*, and *Bacteroides* bacteria in their guts but far lower levels of *Lactobacillus* spp. [254]. Well-balanced gut bacteria provide B vitamins like B6, B5, B3, B12, B7, K, tetrahydrofolate, and folate [255].

Inflammatory diseases like RA lower plasma folate. The long-term use of non-steroidal anti-inflammatory medicines, particularly cyclooxygenase blockers, inhibits vitamin B6 metabolism, lowering blood pyrophosphate [255]. Probiotics release several short-chain fatty acids and vitamins to help nourish the intestinal lumen and lower its pH [256]. *L. casei* ingestion improves rheumatoid arthritis pathology indicators. *Lactobacillus* spp. also hinders the functioning of high amounts of natriuretic and reactive oxygenated species (ROS) in degrading lipids and other macromolecular elements in the affected person’s matrix. *L. casei* reduces joint edema, joint problems, and inflammatory cytokines [257,258].

## 8. Probiotics Preserve Kidney Integrity and Combat Chronic Kidney Disease (CKD)

Chronic kidney disease (CKD) is anticipated to be the fifth major cause of death worldwide, especially in countries with long life expectancies; it could become the second most significant cause of death by the end of the century [259]. Recent studies have shown that finerenone and sodium–glucose co-transporter 2 inhibitors can slow CKD progression, offering promising future prospects. However, renal problems remain, especially in advanced CKD patients [260,261]. Reducing CKD progression and hastened aging requires more therapies. It is well known that various foods affect CKD and may cause acute kidney damage (AKI). Ingesting high levels of oxalate, phosphate, protein, and salt can accelerate CKD, while oxalate can cause acute renal damage [262,263,264].

*L. casei* Zhang protects renal function in mice models of AKI and CKD and in humans. Orally administrating L. acidophilus or *L. casei* Zhang to mice with ischemia-reperfusion injury (IRI) protected against AKI. Compared to L. acidophilus, *L. casei* Zhang enhances renal function, reduces fibrosis-related gene expression, and decreases kidney histological tubular injury. Prebiotics reduced kidney fibrosis in a subtotal nephrectomy model. *L. casei* Zhang lowers macrophage factor expression in the kidneys. *L. casei* Zhang’s beneficial effects remain unaffected by the gut microbiota, even during antibiotic disturbance in clinical settings, especially ICUs [265]

*Lactobacillus casei* Zhang improves renal ischemia/reperfusion (IRI) gut microbiota imbalances. Short-chain fatty acid (SCFA)-producing bacteria, especially Bacteroidetes, proliferate more after this intervention. After IRI induction, probiotics increase the expression of kidney and serum SCFAs such as propionate, butyrate, and acetate. SCFAs also reduce IRI and folic acid-induced nephropathy [266,267]. The intraperitoneal injection of butyrate, acetate, and propionate before ischemia and during reperfusion increases renal health associated with IRI. This improvement is because of the reduced histone deacetylase activity [266]. The oral delivery of SCFAs via drinking water reduces tubular injury caused by folic acid and alleviates interstitial fibrosis and chronic inflammation. Moreover, SCFAs activate receptors, namely Hydroxycarboxylic acid receptor 2 (GPR109A) and G-protein-coupled receptor 41 (GPR41), which protect the kidneys of mice lacking G-protein-coupled receptors in [267].

Moreover, CKD increases gastrointestinal tract urea and ammonium levels. As urea and ammonium levels rise, pH rises, promoting aerobic bacteria growth. Thus, aerobic microflora produce uremic toxins such as trimethylamine N-oxide, indoxyl sulfate, and phosphatidylcholine (PCS), which reduce beneficial anaerobic bacteria in the gut. These facts imply that butyrate-producing microbe reduction contributes to CKD inflammation and progression (Table 2) [268,269]. *L. plantarum* A7-supplemented soy milk significantly reduced oxidized glutathione buildup in people with diabetes with high proteinuria [270].

Probinul neutro^®^ (CadiGroup, Rome, Italy) significantly decreases plasma p-cresol and moderately alleviates gastrointestinal symptoms [271]. Ingesting synbiotics decreases serum p-cresyl sulfate. Although the impact of synbiotics on indoxyl sulfate levels is minimal, the stool microbiota improves with the course of ingesting synbiotics [272]. Lactobacillales and Bifidobacteria markedly alter the gut microbiota and intestinal bacteria metabolism [200]. Probiotics alleviate fecal problems because they biosynthesize lactic acid, hinder the synthesis of toxic compounds by unwanted microbes, and protect kidneys (Table 2) [273].

*B. bifidum* (VDD088), *B. longum* subspecies *infantis* (BLI-02), and *L. acidophilus* significantly decrease blood urea nitrogen and creatinine with varying probiotic doses. High-dose probiotics protect the inflammatory characteristics of the glomerular corpuscles, renal cortex, and healthy renal pelvis from typical compact renal tubules [274]. In CKD, a protein-deficient diet with prebiotics and probiotics improves glomerular filtration rate in patients [275]. In end-stage kidney disease (ESKD) patients, lower levels of *Faecalibacterium* and *Roseburia*, which produce fewer SCFAs, prevail, causing intestinal dysbiosis. ESKD patients possess *Fusobacterium*, *Shewanella*, and *Erwinia*, which are usually absent in healthy individuals. The idea of using gut symbiosis as a CKD treatment is novel and could prove to be effective [276].

### Probiotics Conciliate Kidney Ailments during Hemodialysis and Peritoneal Dialysis

Oral lactic acid bacteria lower bloodstream uremic toxins, particularly indican, in uremia patients. Fecal p-cresol levels decrease significantly in hemodialysis (HD) patients, although plasma p-cresol decreases slightly. Intestinal microbiota also repair suppressed bacterial formation. Probiotic strains reduce plasma indoxyl sulfate and slightly affect indoxyl glucuronide [277]. After ingesting oligofructose-enriched inulin, a great prebiotic, blood PCS levels decrease significantly, whereas IS levels moderately decrease [278]. In HD patients, resistant starch reduces blood indoxyl sulfate levels and also affects serum p-cresyl sulfate levels [279]. Given the information mentioned earlier, it is evident that adding prebiotics to one’s diet is an excellent way to avoid kidney-related diseases. These prebiotics provide nutrition directly to humans and act as a food source for probiotics. Therefore, caution is advised when selecting a probiotic therapy for individuals with HD.

In humans, probiotics reduce the expression of IL-6 and high-sensitivity C-reactive protein. Probiotics significantly improve triceps skinfold thickness, upper arm circumference, and blood albumin in peritoneal dialysis (PD) patients. Therefore, for PD patients, probiotic supplementation improves malnutrition and health [199].

Probiotics reduce proinflammatory cytokines such as tumor necrosis factor-a, IL-5, IL-6, and endotoxins. Probiotics also increase serum IL-10, an anti-inflammatory cytokine, preserving renal function in PD patients [280].

## 9. Probiotics Influence the Microflora of the Male and Female Reproductive Systems

Multiple studies have discussed the bacteria found in the female and male reproductive tracts. Semen contains most male reproductive microorganisms [281]. In contrast, females have microbiomes throughout their reproductive systems, with each tissue or organ colonized by a unique microbiota [282]. Pieces of evidence mark reproductive microbes as crucial for reproductive health and the development of associated illnesses. Commensal bacteria maintain ecological homeostasis in the reproductive tract, improving host fertility and fitness (Table 2) [283]. Dysbiosis in the reproductive microbiome can disturb normal reproductive physiology, causing several pregnancy complications [284]. Given the microbiome’s role in reproductive health and related diseases, probiotic therapies that target the microbiome as a therapeutic approach are rational. Due to the link between metabolic health and reproductive performance, probiotics may improve host reproductive function by modulating metabolism.

### 9.1. Probiotics Enhance Cell Membrane Integrity and Functioning

Probiotics and their bioactive components improve epithelial barrier function. Blastulation, placenta, chorion, and amnion development depend on cellular membrane integrity. Consequently, probiotic strains influence reproductive membrane architecture [285]. Numerous studies have shown probiotics’ immunomodulatory effects. Probiotic strains that alter the inflammatory cascade can yield benefits in terms of reproductive functions and alleviating illnesses related to the reproductive system [286]. Probiotics improve reproductive system performance, reduce illnesses related to the reproductive systems of males and females, and contribute to offspring well-being (Table 2).

### 9.2. Probiotics Conserve Male Reproductive Health

Seminal fluid contains a varied microbiota that protects male reproductive health. A link between serum bacteria and sperm quality exists. In semen, lactobacilli dominate, preserving sperm motility and viability [287]. Thus, semen with a majority of *Lactobacillus* bacteria is of superior quality than semen with other bacteria. Microorganisms can directly cling to sperm and affect spermatozoa. They also inhibit sperm motility indirectly through their metabolites (Table 2) [288].

#### 9.2.1. Probiotics from the Vagina Protect Spermatozoa

Antioxidant enzymes, which protect somatic cells from free radicals, are rare in the cytoplasm of human spermatozoa. In the female reproductive system, spermatozoa may be more susceptible to reactive oxygen species (ROS), especially in infections. The absence of seminal plasma, which contains non-enzymatic antioxidants to protect against oxidative stress, causes susceptibility. Infertile spermatozoa produce excessively high ROS levels, which causes significant peroxidative damage. Sperm membranes with more polyunsaturated fatty acids (PUFAs) are more sensitive to lipid peroxidation [289,290]. A specific mix of three lactobacilli strains (*L. plantarum* FV9, *L. salivarius* FV2, and *L. brevis* CD2), usually used for treating bacterial vaginosis, prevent ferrous ion-induced sperm lipid peroxidation. Hence, this mix protects sperm viability and motility (Table 2). It appears that vaginal probiotic lactobacilli protect human spermatozoa from radical oxygen species during vaginal infections [289].

#### 9.2.2. Probiotics Meliorate Reproductive Hormone Release

Probiotics increase follicle-stimulating hormone (FSH), luteinizing hormone (LH), and testosterone levels, which increases the percentage of progressively motile sperm and velocity characteristics (VCL, curvilinear velocity; VSL, straight-line velocity; VAP, average path velocity), while immotile sperm decreases. Probiotic administration increases the amplitude of lateral head displacement (ALH), linearity (LIN), straightness (STR), and beat cross frequency (BCF). *L. rhamnosus* PB01 is an effective weight loss and reproductive hormone agent, dramatically enhancing kinematic metrics and sperm motility [291]. Probiotics improve spermatogenesis, seminiferous tubule cross-sectional profiles, and testosterone levels, thereby increasing testicular function and semen quality. The systemic treatment of antibodies improves testicular mass and other age-related markers to youthful levels by suppressing the pro-inflammatory cytokine IL-17A. Probiotics mitigate low blood testosterone levels, which have several harmful effects. *L. reuteri* or other probiotic supplements prevent male hypogonadism naturally, avoiding conventional treatments’ controversies and side effects and offering practical solutions for aging-related diseases. Probiotic treatment improves public health by increasing hormonal and gonadal traits linked with reproductive fitness in younger and healthier people [292]. Therapy with *B. longum* CECT7347 and *L. rhamnosus* CECT8361 also increases sperm motility.

#### 9.2.3. Probiotics Attenuate DNA Damage, Blood–Testis Barrier, and Spermatozoa Functionality

Probiotics also significantly reduce DNA fragmentation. Probiotics have been shown to reduce intracellular H_2_O_2_ by a considerable margin. Probiotics preserve cell viability. Moreover, probiotics may improve sperm motility, DNA fragmentation, and ROS in asthenozoospermic males [293]. Probiotics regulate testicular function and blood–testis barrier (BTB) permeability. Probiotics also improve rabbit semen quantity and quality. When mated with nitrate-supplemented bucks, these rabbits have larger litters and heavier babies. These findings imply that probiotics have anti-sterility and offspring-boosting effects [294,295]. Based on substantiated evidence, probiotics have the potential to open up fascinating treatment approaches for addressing infertility.

Research shows that *Bacteroidetes longum* CECT7347 and *L. rhamnosus* CECT8361 improve sperm motility, DNA fragmentation, and intracellular H_2_O_2_ levels. These probiotic strains improve sperm quality due to their antioxidant characteristics [293].

*Lactobacillus casei* significantly reduces *P. aeruginosa*-induced sperm motility and mitochondrial activity, implying that *Lactobacillus* improves semen quality by reducing harmful bacteria. Moreover, probiotic supplements also affect testicular function and spermatogenesis by altering the gut microbiota and its antioxidant characteristics [296,297]. *Bacteroidetes*, *Deferribacteres*, and *Firmicutes* are significantly correlated with diethylhexyl phthalate (DEHP)-induced testicular dysfunction. *L. plantarum* TW1-1 pretreatment reduces testicular damage by modulating microorganisms and reduces testicular injury in DEHP-exposed conditions. Therefore, probiotic strains enhance spermatozoa functionality by altering gut microbiota adaptability. It is also well known that oxidative stress, which probiotic interventions could alleviate, damages sperm DNA and impairs spermatozoa functionality [298].

#### 9.2.4. Probiotics Attenuate Prostatitis and Modulate Lactic Acid Production

The effects of probiotics on the prostate have been narrowly studied in recent years. *B. animalis* Bb-12, *L. casei*-01, *L. acidophilus* La-05, and *L. rhamnosus* GG induce apoptosis in prostate cancer cells [299,300]. Probiotics can also prevent and treat Enterobacteriaceae-induced chronic bacterial prostatitis (Table 2) [301]. Furthermore, probiotics reduced *Enterococcus faecalis* and *E. coli* in prostatitis patients’ urine cultures in [302].

Lactic acid, a “postbiotic” metabolite with antibacterial and immunomodulatory properties, can solve probiotic strain colonization and regulation stability issues. Most eubiosis-associated vaginal bacteria produce more lactic acid [303]. Therefore, lactic acid may restore vaginal microbiome function without needing new and potentially under-evaluated probiotics.

The follicular fluid, which affects oocyte maturation, follicle growth, oviduct transit, steroidogenesis, and ovulation, possesses microorganisms found in the oral mucosa (*Streptococcus* spp.), skin (*Staphylococcus* spp.), gastrointestinal system (*Bifidobacterium* spp., *enteric* bacteria, *Streptococcus agalactiae*), and vagina (*Lactobacillus* spp.) [304]. *Lactobacillus* species dominate follicular fluids and impact embryo maturation and quality. Due to its antibacterial properties, Lactobacilli produce lactic acid to protect oocytes from harmful microorganisms during maturation [305]. The vaginal microbiota of reproductively active women contains *L. gasseri*, *L. crispatus*, *L. jensenii*, and *L. iners*, which produce lots of lactic acid, contributing to eubiosis [303,306,307]. Lactic acid content is inversely correlated with pH in women with *Lactobacillus*-dominated microbiota, suggesting its role in vaginal acidification [308].

### 9.3. Probiotics Alleviate Bacterial Vaginosis (BV)

The lower and upper reproductive tracts make up the female reproductive system. The reproductive organs—the uterus, fallopian tubes, and ovaries—are in the upper part, while the vagina and cervix are in the lower. Both the lower and upper reproductive tracts have microflora. There was formerly a belief that the upper system was microbial-free. Recent research has confirmed the presence of microflora in the placenta, fallopian tubes, follicles, and uterus [283,309,310]. Lower reproductive tract microbiomes are diverse and abundant. Each region owns a microbiome with a unique makeup and diversity. Age, physiological conditions, lifestyle, and environment affect reproductive tract microflora composition [284,288,311].

Little is known about the etiology and pathogenesis of bacterial vaginosis (BV), a common infection in reproductive-age women with harmful sexual and reproductive health effects [312].

The dominant attributes of probiotics in treating BV include synthesizing antimicrobial compounds such as bacteriocins and H_2_O_2_, adhering to vaginal epithelial cells, and having antimicrobial effects. Probiotics also acidify the vaginal environment by producing lactic acid, which has immunomodulatory effects. Moreover, probiotics outcompete unwanted bacteria, co-assemble, and resist antibiotics, excluding those used to treat BV [313,314,315,316]. Many *Lactobacillus* strains and species, including *L. fermentum*, *L. reuteri* RC-14, *L. gasseri*, *L. brevis*, *L. plantarum*, *L. acidophilus*, *L. rhamnosus* GR-1, and *L. crispatus*, are effective as vaginal probiotics for BV treatment or prevention (Table 2) [313,315,317].

Lactic acid alone, without bacteriocins, kills BV-associated bacteria in vaginal secretions in conditions that mimic ex vivo conditions. Lactal, a lactic acid gel used for BV recurrence prevention and therapy, results in a significant clearance rate for bacterial vaginosis without exhibiting deleterious effects. Treatment with Lactobacilli restores colonization in the lower female reproductive tract [318]. Lactic acid gels and other lactic acid sources re-colonize lactobacilli after a few days [319,320].

### 9.4. Products from Probiotics Hinder Sexually Transmitted Infections (STIs)

Lactic acid fights bacterial STIs better than hydrogen peroxide. *L. gasseri* and *L. crispatus* form lactic acid to inactivate *E. coli*, *Neisseria gonorrhoeae*, and *Chlamydia trachomatis* [321,322,323,324]. Lactic acid from *L. crispatus* inhibits bacterial growth in live tissue. A porcine vaginal mucosa model suppressed *Gardnerella vaginalis* and *N. gonorrhoeae* [325]. Lactic acid, directly or via a probiotic strain, helps to maintain eubiosis or restore dysbiosis in vaginal microorganisms, preventing bacterial STIs. Hydrogen peroxide-producing lactobacilli in women reduce dysbiotic microbiota. *Lactobacillus* spp., especially *L. crispatus*, exists in the vaginal microbiota and produces hydrogen peroxide in aerobic environments, establishing eubiosis [324].

### 9.5. Probiotics Countermine HIV and Herpes Simplex Virus 2 (HSV-2)

Women with a dominant lactobacillus vaginal microbiome are less likely to contract HIV from males. Furthermore, HIV-infected women with a lactobacillus-dominated microflora release fewer viral particles into the lower female reproductive tract [326,327]. This intervention protects males and vaginally delivered neonates from sexually transmitted HIV. Physiological concentrations of lactic acid inactivate HIV faster and more effectively than medium acidified to the same pH with acetic acid or HCl [328].

BV is also a good predictor of HSV-2. Women with a lactobacilli-dominated vaginal microbiota are less likely to contract HSV-2 [327]. Furthermore, vaginal lactobacilli and their products strongly inactivate STIs. *Lactobacillus* species inhibit HSV-2 in virucidal-independent and virucidal-dependent ways. Due to lactic acid or lactobacilli adhesion, the virucidal-independent processes suppress viral entry and reproduction. An acidic pH affects lactic acid’s HSV-2 inhibition. These facts suggest that protonated lactic acid mediates the effect [329,330,331].

### 9.6. Probiotics Improve Ovarian Function

Few studies have examined how probiotic strains affect follicular formation in women. Probiotics may delay ovarian function and estradiol decline in menopausal women to reduce menopause symptoms like dyslipidemia and obesity. Probiotics from healthy women raise estrogen levels in ovariectomized menopausal mice due to gut microflora’s versatile metabolites, suggesting that probiotic intervention regulates estradiol levels in ovarian failure. Perimenopausal women can preserve ovarian function by taking the probiotic Sanprobi Barrier, which raises FSH levels; a non-invasive intervention that regulates hormonal balance is possible [332,333]. Probiotic strains boost avian follicular growth. In Hy-Line layers, Bacillus improves egg bulk and production [334,335], and *Enterococcus faecium*, after being included in AA broiler breeders’, diets increased egg weight and shell thickness. Probiotics increase reproductive hormones like FSH, estradiol, and growth hormone and reduce adrenal cortical hormone levels, regulating follicle development [335,336]. Probiotics also improve fish follicle development. *L. rhamnosus* IMC 501 alters zebrafish oocyte constitution, facilitating maturation. Probiotics influence the endocrine system and peripheral tissues to upregulate leptin and Kiss2 and Kiss1 gene expression, modulating the constitution and maturation of oocytes [337].

### 9.7. Probiotics Maintain the Steady Provision of Crucial Elements through the Placenta

The transient placenta connects the mother’s uterus to the developing fetus during gestation. The placenta transfers essential nutrients and oxygen from the mother to the developing fetus, ensuring normal fetal growth [338]. Oral probiotics influence placental function. Probiotics such as *E. faecium*, especially its genetically modified strain, can migrate from the gastrointestinal tract to the developing fetus’s amniotic fluid via the placenta [339], suggesting that probiotic diets may influence the placental microbiome and placental functionality. Probiotics regulate placental genes associated with Toll-like receptors (TLRs) and autophagy-related proteins [340,341]. In infant–mother couples, placental tissue samples contain bacterial DNA. Microbial DNA in the placenta and amniotic fluid increases TLR-related gene expression in fetal intestinal tissue. Administering probiotics during pregnancy significantly regulated TLR-related genes in the gastrointestinal tract and placenta. During fetal development, microorganisms alter innate immunity gene expression in the intestines, implying that a maternal nutrition intervention with targeted probiotics may alter fetal and placental immune physiology [340]. *L. rhamnosus* GR-1 inhibits TNF-α generation in human placenta trophoblast cells induced by lipopolysaccharides (LPSs). Lipopolysaccharide administration increased IL-10, TNF-α, and prostaglandin-endoperoxide synthase 2 (PTGS2) expression, with a more significant prevalence in male placentae. The *L. rhamnosus* GR-1 supernatant hinders TNF-α production from LPS and promotes IL-10 production. The female placentae expressed more PGDH, while the male placentae had less LPS-stimulated PTGS2 and more TLR-4. These findings support lactobacilli as a treatment for premature labor [342]. Probiotics reduce placenta inflammation, reducing the risk of severe preeclampsia. First-time mothers who regularly consume milk-derived probiotics are less likely to develop preeclampsia [343]. Probiotics affect placental function by boosting immune response. Prebiotics and probiotics also increase sow serum triacylglycerol and decrease umbilical venous serum total cholesterol, suggesting that probiotics and prebiotics improve placental lipid metabolism [344]. These findings show that probiotic interventions improve placental function by changing microbiota composition, increasing immune response, and improving metabolic control during pregnancy.

Probiotic supplementation improves gut microflora and metabolism in pregnant women, elevating the well-being of pregnant women and the fetus [345,346]. In meconium composition, when ingested during pregnancy, *B. lactis* and *L. rhamnosus* probiotics affect TLR gene regulation in the developing gut of the fetus, suggesting that probiotic strains affect the fetus’ immune system [340]. Probiotics, as placental therapies, also have the potential to reduce preterm delivery and placental efficiency [347]. Probiotic supplements in the later stages of pregnancy significantly elevate birth weight and litter weight, regardless of the round of pregnancy. Probiotics affect fetal development by increasing feed intake and immunoglobulin levels and modulating gut flora [348,349,350]. In umbilical venous serum, *Bacillus* mixed with isomaltooligosaccharide, a prebiotic, increases placental antioxidant capacity and growth hormone levels, leading to enhanced fetal development [344].

## 10. Probiotics Preclude the Prevalence of Cardiovascular Disease (CVD)

Over the past few decades, cardiovascular disease (CVD) has caused the most premature death and disability in low- and middle-income countries. Most developed nations have over 50% of middle-aged fatalities and 33% of senior deaths due to CVD. Cardiovascular disease (CVD) encompasses many cardiovascular system abnormalities, including peripheral vascular disease, cerebrovascular disease, and coronary heart disease (CHD). These conditions affect blood flow to the heart, brain, and peripheral organs [351]. Lesions in coronary, cerebral, or peripheral arteries can cause CVDs, leading to atherosclerosis, thrombosis, and clots [352,353].

Immune responses play a significant role in the development of atherosclerosis. Lipid plaques characterize atherosclerosis in arterial walls that gradually grow. Cholesterol from bloodstream LDL particles makes up these plaques. Lipoproteins enter arterial walls’ subendothelial compartment, activating endothelial cells. Monocytes in the arterial wall differentiate into macrophages, internalizing lipoproteins and becoming foam cells, a hallmark of atherosclerotic plaque [354]. Lipid-driven atherosclerosis, a chronic disease causing inflammation, is a major risk factor for heart disease and stroke. Clotting is a common pathophysiological mechanism in CVD, which begins with an inactive precursor or zymogen and then involves a cascade of proteolytic processes [351].

### 10.1. Probiotics Regulate Plasma Glucose and Inulin Levels

Probiotic supplementation reduces fasting plasma glucose, insulin resistance, insulin, and serum high-sensitivity C-reactive protein and elevates glutathione and antioxidant capacity. Moreover, probiotics improve total-/HDL-cholesterol ratio, HDL-cholesterol, glycemic control, oxidative stress, and inflammation in diabetic CHD patients (Table 2) [355].

The homeostasis model shows that vitamin D and probiotics reduce serum insulin levels and insulin resistance. Additionally, serum 25-OH-vitamin D levels, HDL-cholesterol, and quantitative insulin sensitivity check index elevate. Probiotic interventions significantly influence the plasma total antioxidant capacity (TAC), plasma nitric oxide (NO), and serum high-sensitivity C-reactive protein (hs-CRP) levels. Vitamin D and probiotics co-administration in diabetics and CHD patients improves mental health, serum hs-CRP, plasma NO, TAC, glycemic management, and HDL cholesterol [356].

Probiotics and selenium reduce fasting plasma insulin resistance, serum insulin levels, glucose levels, and insulin sensitivity. Co-supplementation also significantly reduces hs-CRP (Table 2), very low-density lipoprotein (VLDL), total cholesterol, and triglycerides and increases serum NO, total glutathione, and total antioxidant capacity. Selenium and probiotic supplements improve metabolic health in diabetics and CHD patients [357].

Synbiotic capsules reduce fasting plasma glucose and serum insulin levels. The intervention decreases the homeostasis model of b-cell function, elevates the quantitative insulin sensitivity check index, and significantly changes HLDL-cholesterol changes. The administration of synbiotic supplements to diabetic and CHD patients improves insulin metabolism and HDL cholesterol [358].

### 10.2. Probiotics Engage as Comforters in Coronary Artery Disease (CAD)

*Lactobacillus plantarum* 299v, as a circulatory system comforter, significantly improves brachial flow dilation but moderately influences body mass index, fasting glucose, and plasma cholesterol. *L. plantarum* 299v supplementation also decreases circulation levels of leptin, IL-12, and IL-8; however, it minimally affects plasma trimethylamine oxide. Moreover, the intervention raises propionate levels in plasma while reducing acetate. In CAD patients, *L. plantarum* 299v plasma increases endothelium-dependent vasodilation, improves vascular endothelial function, and reduces systemic inflammation in male CAD patients regardless of trimethylamine oxide levels or risk factors [359].

*Lactobacillus reuteri* also reduces myocardial injury after ischemia/reperfusion (I/R). *L. reuteri* ingestion protects against heart damage regardless of cholesterol levels, demonstrating the anti-inflammatory effects of probiotics without cholesterol benefits. Daily *L. reuteri* administration to normal and hypercholesterolemic lipoprotein receptor deletion mice decreases myocardial damage following ischemia-reperfusion without lowering total serum cholesterol. *L. reuteri* ensures cardiac damage protection and reduces ischemic heart injury as a probiotic [360].

### 10.3. Probiotics Diminish Inflammation-Associated Ailments

In cardiac injury patients, probiotics lower peripheral inflammation and boost FoxP3+, CD25+, and CD4+ regulatory T cells (Tregs) [361,362]. Congestive heart failure (CHF) reduces Tregs. Tregs at a low level leads to a poor prognostic approach in CHF patients with vitiated cardiac functioning [363].

*Bifidobacterium animalis* subsp. *lactis* 420, a potent probiotic, reduces heart inflammation. Specifically, it reduces heart damage from ischemia/reperfusion and causes left coronary artery permanent closure. Probiotics lead to Treg cell activation and epigenetic changes. Probiotics possess various therapeutic benefits for human diseases; however, extending their findings to comprehensive clinical cardiovascular protection is tricky [364].

Probiotics reduce peripheral inflammation by converting gut and peripheral dendritic cells into Treg [361]. Gut metabolites from microflora target conserved non-coding sections of foxp3 genes to affect Tregs directly. This interaction can increase FoxP3 acetylation, improving Treg cell activity and expression [365,366]. *B. animalis* subsp. *lactis* 420, elevates Ac-H3, normalized compared to H3, and increases posttranslational and epigenetic remodeling [364]. *L. rhamnosus* GR-1 shows excellent potential as a therapy for reducing the severity of heart failure [367]

## 11. Probiotics Alleviate Neurodegenerative and Neurodevelopmental Disorders

Probiotics and vitamin D improve psychological measures, as they have been shown to prompt decreases in Beck Depression Inventory, General Health Questionnaire, and Beck Anxiety Inventory scores [356]. Moreover, probiotic and selenium co-administration significantly reduced the Beck Anxiety Inventory index and Beck Depression Inventory score (Table 2) in [357].

Psychobiotics are probiotics with mental health benefits which produce or induce anti-inflammatory cytokines, SCFAs, neurotransmitters, and enteroendocrine hormones. They reduce stress and mood and aid in treating neurodegenerative and neurodevelopmental problems. The most prevalent psychobiotics are Enterococci, Streptococci, Lactobacilli, Escherichia, and Bifidobacteria. These bacteria regulate the gut–brain connection. Gut bacteria biosynthesize substances that enteric nervous system neurons use to communicate with the CNS [368].

Psychobiotics can treat various neurological illnesses, from stress, anxiety, and mood swings to Parkinson’s and Alzheimer’s (Table 2). Chronic psychobiotic use normalizes anxiety and depressive behavior [369,370]. *B. longum* 1714 strain improves cognition, behavior, and physiological response. *L. rhamnosus* JB 1 reduces despair-induced corticosterone and raises plus maze anxiety [371,372,373].

### 11.1. Probiotics Combat Insomnia

Psychobiotics have tremendous potential to treat insomnia and can improve Non-Rapid Eye Movement sleep efficiency and reduce awakening episodes in insomniacs during the resting period [374,375].

### 11.2. Probiotics Assist in Accommodating for Autism Spectrum Disorder (ASD)

Psychobiotics fix the dysbiosis detected in ASD patients, as gut microbiota, including *Clostridium*, *Prevotella*, *Firmicutes*, and *Bacteroidetes*, change during ASD [376,377]. The composition of SCFAs in the stool samples of people with ASD differs, but the relevance of these variations concerning autistic symptoms has only been narrowly explored. Moreover, metabolic products from probiotics butyrate improve ASD symptoms [378]. Neuropsychiatric disorders, including schizophrenia, are associated with a specific neurotransmitter, dopamine, which is synthesized by microflora [379], suggesting that alterations in the gut microbiota have an association with the development of schizophrenia.

### 11.3. Probiotics Help in Coping with Attention Deficit Hyperactivity Disorder (ADHD)

Psychobiotics also aid those with ADHD and Tourette’s syndrome who experience involuntary vocalizations and movements called ‘tics.’ [379]. Pieces of evidence demonstrate that the CNS and gut microbiota are linked to this effect, since ADHD risk factors are directly linked to gut microflora changes [380].

### 11.4. Probiotics Temper Parkinson’s and Alzheimer’s Disease

Environmental factors such as the gut microbiota are major players in developing neurodegenerative disorders like Parkinson’s disease, as shown through the changes in bowel function that occur before the typical motor symptoms appear. Dysbiosis in such a situation creates abnormal levels of particular bacteria, including *Proteobacteria* of the genus Ralstonia, *Faecalibacterium*, *Enterobacteriaceae*, *Prevotellaceae*, and butyrate-producing bacteria with ‘anti-inflammatory’ properties [378]. Additionally, patients with Parkinson’s disease exhibit reduced levels of SCFAs, indicating their potential involvement in the progression of diseases [381].

Furthermore, manipulating the gut microflora can have a positive impact on microglial activation and neuronal function in Alzheimer’s disease (AD) (Table 2). The risks for AD, such as obesity and type 2 diabetes, have an influence on the constitution of the microflora [378]. Changes in the population of microorganisms result in increased intestinal permeability and systemic inflammation, which, in turn, can lead to diabetes mellitus and insulin resistance [382]. The pathophysiology of AD is characterized by the accumulation of misfolded amyloid proteins. These proteins undergo sequential cleavages by various proteases, resulting in the formation of the Aβ peptide. The gut microbiota regulates protease enzymatic activity, leading to inflammation. The gut microbiota is also a major element in the buildup of amyloid plaques [383]. These findings demonstrate psychobiotics’ potential as supplements to traditional drugs that can be employed to treat neuropsychiatric and neurodevelopmental diseases and for broader uses in the field.

## 12. Conclusions and Future Prospects

Probiotics exhibit considerable potential in promoting health and are frequently employed as agents that modulate the gastrointestinal tract to improve overall human health. The host possesses a significant defense mechanism known as the antioxidant system, as free radicals have been linked to many forms of cellular damage and consequent metabolic diseases or disorders. The therapeutic and prophylactic effects of probiotic microorganisms are attributed to their ability to produce numerous bioactive compounds and release them into the bloodstream through the digestive system or their area of prevalence, such as the oral and vaginal cavities. This is mainly achieved by forming short-chain fatty acids (SCFAs). These SCFAs serve as potent agents against several ailments and toxic conditions. Certain strains of probiotics can metabolize toxic chemicals, particularly amines and N-nitroso compounds. Short-chain fatty acids (SCFAs) and other bioactive compounds are produced in the colon through fermentation and delivered to the diseased areas through the bloodstream. These compounds elicit therapeutic effects on the host through a multitude of mechanisms, including alterations in the metabolic activity and composition of the gut microflora, maintaining intestinal health by strengthening the gut barrier and mucus layer, immunomodulation, the degradation and binding of toxic compounds, the modulation of the expression levels of genes in different organs, the altering of pathogen functioning, changes in host physiology, the inhibition of cell proliferation, anti-mutagenic effects, and the induction of apoptosis in cases involving cancer.

Moreover, it has been established that many probiotics, such as *Lactobacillus rhamnosus* GG, influence several organs simultaneously. For instance, *L. rhamnosus* GG palliates oral and gastrointestinal disorders, improves barrier dysfunction, reduces diarrheal episodes, treats hypertransaminasemia, reduces pancreatic cancer risk, reduces pathogenic Proteobacteria, exhibits beneficial effects on cardiac remodeling, and exerts healthful impacts on inflammatory biomarkers, anxiety, depression, etc. *L. rhamnosus* GG achieves this by promoting the production of anti-inflammatory cytokines such as interleukin (IL)-4 and IL-10, hindering pro-inflammatory cytokines, including IL-1, IL-6, and TNF-α. Several *Lactobacillus* spp. and *Bifidobacterium* spp. have the curative power needed to soothe several organs, requiring robust exploration. Furthermore, a sprucely designed cocktail of *Lactobacillus* and *Bifidobacterium* species can alleviate ailments throughout the human body, diminishing the need for multiple doses or medication. 

The use of probiotics over an extended period has the potential to enhance and regulate the immune system by inflecting the related regulatory genes and releasing anti-inflammatory cytokines. Furthermore, incorporating food supplements containing a synergistic blend of appropriate probiotics has the potential to augment the functionality and viability of the host organism. Investigating the modification of the regulation of the immune system, gut microflora, and other pertinent pathways presents a captivating direction for further exploration. Furthermore, studying the influence of probiotics on receptors within human cells and their coordination with other organs is essential. These investigations could specifically aim to determine whether these effects are directly caused by probiotic interventions or whether alterations in the overall constitution of the microflora arbitrate them. Such research is crucial for comprehending the mechanisms elucidated in this review article.

Furthermore, the need for alternative therapeutics can be better understood by examining the escalating rate of antibiotic resistance in case of bacterial infections and the declining patient adherence to current treatment protocols. Therefore, while the antagonistic effects of probiotics on several pathogens are specific to certain strains, the potential for probiotics to be utilized as a future treatment modality, either alone or in conjunction with established therapeutic approaches such as adjuvant therapy, drug delivery systems, and immune system enhancement, may increase in light of the ongoing and anticipated rise in antibiotic resistance.

Hence, forthcoming investigations may need to contemplate conducting in vitro examinations on synthesizing probiotic bioactive compounds that exhibit gastrointestinal properties within a simulated gastrointestinal environment encompassing a combination of enzymes, acids, salts, mucus, and other relevant factors. To accurately determine the genes and bioactive chemicals that are activated and produced in eaten probiotics, it is imperative for such research to also take into account the impact of illness circumstances on the immune system, microbial competition, and gut host antimicrobial proteins.

It is imperative to conduct meticulously designed trials to enhance our knowledge and comprehension of the specific probiotic molecules that elicit particular effects. These trials should employ appropriate quantities of purified bioactive chemicals derived from probiotics or suitable numbers of probiotic mutants with targeted gene knock-outs or knock-ins. These challenges would necessitate the establishment of various crucial parameters, including the potency of the probiotic strain, its optimal dosage, the desired host response, its specific place within the host organism, and other pertinent factors.

## Figures and Tables

**Table 2:** Alleviative effects of probiotics on the respiratory system, bone health, kidneys, cardiac health, and reproductive system, as established in placebo-controlled human studies.

Name of the Probiotic Strains	Age of the Participants	Dose of the Probiotics	Duration of Study	Outcomes of the Study	References
Probiotics Fortify the Respiratory Tract					
*Lactobacillus acidophilus*, *Lactobacillus casei*, *Lactobacillus bulgaricus*, *Lactobacillus rhamnosus*, *Streptococcus thermophilus Bifidobacterium longum*, *Bifidobacterium breve*,	9–53 years	1 × 10^9^ CFU/g	60 days	Slightly increased expression of forkhead box P3 (FoxP3); transforming growth factor-β (TGF-β) and interferon-gamma (IFN-γ); and decreased interleukin (IL)-10 and IL-4 expression. Significantly decreased IL-17 values after synbiotic intake, showing controlled immunopathogenesis of allergic rhinitis.	[183]
*Bifidobacterium breve M-16*, *Bifidobacterium infantis M-63*, *Bifidobacterium longum BB536*	4–17 years	1 × 10^9^, 1 × 10^9^, 3 × 10^9^ CFU/g	56 days	Significantly elevated quality of life (QoL) and alleviated nasal symptoms in children with allergic rhinitis.	[184]
*Lactobacillus acidophilus*, *Lactobacillus casei*	18–21 years	-	28 days	Significantly elevated IFN-γ levels and decreased IL-4 levels, and significant difference among the IFN-γ:IL-4 exhibiting improved IFN-γ/IL-4 levels in patients with allergic rhinitis.	[185]
*Bifidobacterium lactis* NCC2818	20–65 years	2 × 10^9^ CFU/g	56 days	Significantly lowered Th-2 cytokine concentrations (IL-3 and IL-5), nasal symptom scores, and activated-CD63-expressing basophils, exhibiting the capacity of NCC2818 to mitigate allergic symptoms and immune parameters.	[186]
*Lactobacillus Paracasei* 33	0.5–5 years	2 × 10^9^ CFU/g	42 days	Significantly improved nasal blocking, sneezing, rhinorrhea, feeding and sleeping difficulties, and coughing, showing the equal effectiveness of *Lactobacillus paracasei* 33 as cetirizine in children with perennial allergic rhinitis without any significant side effects.	[187]
*Bifidobacterium breve* B632 *Ligilactobacillus salivarius* LS01	3–14 years	1 × 10^9^ CFU/g each	112 days	The significant reduction in asthmatic exacerbations demonstrates the effectiveness and safety of *Bifidobacterium breve* B632 and *Ligilactobacillus salivarius* LS01, proving that they are auxiliary remedies.	[188]
*Lactobacillus acidophilus*, *Lactobacillus rhamnosus*, *Lactobacillus bulgaricus*, *Lactobacillus casei*, *Streptococcus thermophiles*, *Bifidobacterium longum*, *Bifidobacterium breve*,	38.62 ± 10.49 years	3 × 10^9^, 7 × 10^9^, 5 × 10^8^, 3 × 10^9^, 3 × 10^8^, 1 × 10^9^, and 2 × 10^10^ CFU/g	60 days	Significant reduction in IL-4, miR146-a, and miR-16 levels and elevation in miR-133b level displayed significantly improved forced vital capacity (FVC) and forced expiratory volume for 1s (FEV1).	[189]
*Lactobacillus salivarius* PM-A0006	10–12 years	2 × 10^9^ CFU/g	112 days	Significantly improved pulmonary functioning parameters, such as FVC, FEV1, and FEV1:FVC ratio, and significantly decreased use the of inhaled corticosteroids and short-acting inhaled bronchodilators, as well as the diminished use of medicine in asthmatic children.	[190]
*Streptococcus thermophilus*, *Lactobacillus bulgaricus*, *Lactobacillus acidophilus*, *Lactobacillus rhamnosus*, *Lactobacillus casei*, *Bifidobacterium infantis*, *Bifidobacterium breve*	6–12 years	1 × 10^9^ CFU/g each	60 days	Significantly lowered the number of viral respiratory infections and decreased the use of Salbutamol.	[191]
*Lactobacillus bulgaris*, *Lactobacillus Casei*, *Streptococcus thermophiles*, *Lactobacillus acidophilus*, *Bifidobacterium breve*, *Lactobacillus rhamnosus*, *Bifidobacterium infantis*,	≤12 years	Manufacturer defined recipe	180 days	Significantly alleviated asthma symptoms and decreased outpatient visits with rare side effects, exhibiting positive effects on the QoL of asthmatic patients.	[192]
Probiotics Meliorate Bone Health					
*Bacillus subtilis* C-3102	50–69 years	3.4 × 10^9^ CFU/g	168 days	Significant increase in the *Bifidobacterium* genus and a decrease in the *Fusobacterium* genus. Significant increase in hip BMD and decrease in bone resorption markers such as urinary type I collagen cross-linked N-telopeptide (uNTx) and tartrate-resistant acid phosphatase isoform 5b (TRACP-5b), suggesting improved BMD by gut microbiota modulation and bone resorption inhibition in postmenopausal women.	[193]
*Streptococcus thermophiles*, *Bifidobacterium* *breve*, *Bifidobacterium longum*, *Lactobacillus acidophilus*, *Lactobacillus bulgaricus*, *Lactobacillus rhamnosus*, *Lactobacillus casei*,	50–72 years	3 × 10^8^, 2 × 10^10^, 1 × 10^11^, 3 × 10^10^, 5 × 10^8^, 7 × 10^9^, and 2.6 × 10^10^ CFU/g	180 days	Significant decrease in cross-linked C-telopeptide (CTX) and bone-specific alkaline phosphatase (BALP), TNF-α, and serum parathyroid hormone (PTH) levels.	[194]
*Lactobacillus plantarum* DSM 15313 and DSM 15312, *Lactobacillus paracasei* DSM 13434	59.1 ± 3.8 years	1 × 10^10^ CFU/capsule	365 days	Significantly reduced lumbar spine bone mineral density (LS-BMD) losses.	[195]
Probiotics Preserve Kidney Integrity					
*Bifidobacterium longum* A101 *Bifidobacterium bifidum* A218 *Lactobacillus rhamnosus*, *Lactobacillus Plantarum* A87	22–69 years	1 × 10^9^ CFU/g each	90 days	Significantly decreased serum syndecan-1 and blood glucose levels, indicating improved metabolism and systemic inflammation reduction in chronic kidney disease patients.	[196]
*Lactobaccillus pentosus* LPE588, *Lactobaccillus salivarius* LS159, *Lactococcus lactis* subsp. *lactis* LL358	39–75 years	1 × 10^11^ CFU/g	180 days	Significantly decreased indoxyl sulfate levels in serum in patients on hemodialysis (HD), with slight changes in serum p-cresyl sulfate, blood urea nitrogen, hemoglobin levels, blood glucose, microbial and inflammatory translocation markers.	[197]
*Lactobacillus acidophilus*, *Streptococcus thermophilus*, *Bifidobacterium longum*	≥18 years	9 × 10^10^ CFU/capsule	7 days	Significantly decreased blood urea values.	[198]
*Streptococcus thermophilus*, *Lactobacillus bulgaricus*, *Bifidobacterium longum*	18–75 years	1 × 10^9^ CFU/g	60 days	Significantly decreased high-sensitivity C-reactive protein (hs-CRP) and IL-6 levels and increased triceps skinfold thickness, upper arm circumference, and serum albumin levels in peritoneal dialysis patients, leading to higher social and physical functioning, as well as improved QoL and malnutrition.	[199]
*Lactobacillus rhamnosus* HN-001 and LR-32, *Enterococcus faecium* UBEF-41, *Saccharomyces cerevisiae subspecies Boulardii* MTCC-5375, *Lactobacillus acidophilus* LA-14, *Bifidobacterium longum* BL05, *Bifidobacterium bifidum* BB06, *Bifidobacterium brevis* BB03	>18 years	Manufacturer’s recipe	111 days	Significantly elevated fecal *Bifidobacteria* and *Lactobacillales* concentrations and diminished 3-methyl-indole (3-MI) and urinary indican levels. Significantly improved levels of serum calcium, ferritin, iron, C-reactive protein, transferrin saturation, serum intact parathormone (iPTH), and β2-microglobulin.	[200]
*Lactobacillus casei*, *Bifidobacterium lactis*, *Lactobacillus acidophilus*,	≥18 years	3.2 × 10^10^ CFU/g	84 days	Significantly improved *Subdoligranulum*, *Lactobacillus*, and *Bifidobacteria* genera and glomerular filtration rate. Decreased serum IS and hr-CRP levels, exhibiting decreased microinflammation and uremic toxins levels in patients with chronic kidney disease.	[201]
*Lactobacillus rhamnosus* GG	70.15 ± 12.3 years	3.5 × 10^11^ CFU/g	56 days	Significantly lowered the serum P-cresol sulfate (PCS) and albuminuria and improved estimated glomerular filtration rate (eGFR), serum creatinine (SCr), blood urea nitrogen (BUN), and proteinuria. Significantly reduced pathogenic Proteobacteria and elevated *Firmicutes* and *Actinobacteriota* count, suggesting gut microbiota melioration.	[202]
*Streptococcus thermophilus Lactobacillus acidophilus*, *Bifidobacterium bifidum*,	18–75 years	4.3 × 10^9^, 4.2 × 10^9^, and 1.2 × 10^9^ CFU/g	84 days	Significantly reduced fasting blood glucose, mAlb/Cr, and HbA1c and slightly decreased 2h postprandial blood glucose level and eGFR while somewhat elevating mAlb/Cr levels, suggesting ameliorated glycemic control in diabetic nephropathy patients.	[203]
*Bifidobacterium bifidum*, *Bifidobacterium longum Bifidobacterium lactis*, *Lactobacillus acidophilus*,	30–65 years	2.7 × 10^7^ CFU/g each	84 days	Significantly decreased beck depression inventory (BDI) and beck anxiety inventory (BAI) and significantly increased serum hemoglobin (Hb) levels.	[204]
*Enterococcus faecalis* YIT0072 *Lactobacillus acidophilus* YIT2004, *Bifidobacterium longum*,	18–70 years	1.1 × 10^9^, 0.53 × 10^9^, and 2.2 × 10^9^ CFU/g	180 days	Significantly restored Bacteroidaceae and Enterococcaceae, and reduced Clostridiales Family XIII. Incertae Sedis, Erysipelotrichaceae, Peptostreptococcaceae, Ruminococcaceae, and Halomonadaceae in non-diabetic hemodialysis patients. Significantly reduced uremic retention solutes, including 1-methylinosine, 3-guanidinopropionic acid, and indole-3-acetic acid-O-glucuronide, in feces or serum.	[205]
*Bifidobacterium lactis* BIA-6, *Bifidobacterium longum* LAF*-5*, *Lactobacillus acidophilus* T16, *Bifidobacterium bifidum* BIA-6	30–65 years	2.7 × 10^7^ CFU/g each	84 days	Significantly altered IL-6 and hs-CRP and significantly changed anti-HSP70 after synbiotic intake, exhibiting improved anti-HSP70 serum levels, endotoxin, and inflammatory markers.	[206]
Probiotics Preclude the Prevalence of Cardiovascular Diseases					
*Lactobacillus rhamnosus* GG	56.70 ± 9.10 years	1.6 × 10^9^ CFU/g	90 days	Significantly decreased serum TGF-β and trimethylamine N-oxide (TMAO) levels. Sightly differed matrix metalloproteinase-9 (MMP-9) and procollagen III levels and improved echocardiographic indices. Left ventricular ejection fraction (LVEF) and variation in procollagen III predicted 62% final LVEF levels, exhibiting beneficial effects on cardiac remodeling in myocardial infarction patients.	[207]
*Bifidobacterium lactis*, Bb12, *Lactobacillus acidophilus La-5*	30–70 years	1 × 10^7^ CFU/g each	70 days	Significantly decreased oxidized low-density lipoprotein (ox-LDL) and apolipoprotein B100 (ApoB100) and slightly changed N-terminal pro-brain natriuretic peptide (NT-proBNP) and pentraxin3 (PTX3), suggesting improvements in the oxidative status of congestive heart failure (CHF) patients.	[208]
*Lactobacillus rhamnosus* GG	30–70 years	1.6 × 10^9^ CFU/g	84 days	Significantly decreased low-density lipoprotein cholesterol and total cholesterol, with slight differences in blood pressure and MetS feature indices suggesting decreased cardiovascular risk factors.	[209]
*Streptococcus thermophiles*, *Bifidobacterium longum*, *Lactobacillus acidophilus*, *Lactobacillus casei*, *Lactobacillus bulgaricus*, *Bifidobacterium breve*, *Lactobacillus rhamnosus*,	30–70 years	1.5 × 10^8^, 5 × 10^8^, 1.5 × 10^10^, 1 × 10^9^, 2.5 × 10^8^, 1 × 10^10^, and 3.5 × 10^9^ CFU/g	70 days	Significantly decreased NT-proBNP levels and hindered the elevation of hs-CRP, exhibiting the advantageous effects of synbiotics on inflammatory status.	[210]
*Lactobacillus rhamnosus* GG	8–85 years	1.9 × 10^9^ CFU/g	60 days	Significant reductions in BDI, Spielberger state–trait anxiety inventory (STAI) state, and STAI-trait scores, lipopolysaccharide (LPS), TNF-α, and hs-CRP, suggesting beneficial health impacts on inflammatory biomarkers, anxiety, and depression.	[211]
*Lactobacillus paracasei* LPC-37, *Bifidobacterium lactis* HN019, *Lactobacillus acidophilus* NCFM, *Lactobacillus rhamnosus* HN001	20–50 years	1 × 10^9^ CFU/g each	56 days	Significant reduction in cholesterol and fasting glucose levels while elevating HDL-cholesterol. Slightly lowered systolic BP and diastolic BP and reduced low-frequency (LF) oscillation and LF/high-frequency (HF) ratio, suggesting improved autonomic modulation and lipid profiles in hypertensive women.	[212]
*Lactobacillus rhamnosus* GG	56.70 ± 9.10 years	1.6 × 10^9^ CFU/g	84 days	Significantly decreased IL1-Beta and LPS levels and significantly meliorated cardiovascular-related factors, suggesting advantageous impacts on mega inflammation and metabolic endotoxemia in coronary artery disease patients.	[213]
Probiotics Influence the Male and Female Reproductive Systems					
*Lactobacillus casei* DG	18–45 years	2.4 × 10^10^ CFU/g	90 days	Significantly changed QoL, International Prostate Symptom Score (IPSS), and NIH Chronic Prostatitis Symptom Index (NIH-CPSI) and significantly decreased antibiotic use and symptomatic recurrence.	[214]
*Escherichia coli* Nissle 1917	≥ 18 year	2.5–25 × 10^9^ CFU/g	84 days	Significantly lowered biological recurrence rate and NIH-CPSI score, exhibiting efficiently controlled and diminished biological recurrences in chronic bacterial prostatitis patients.	[215]
*Lactobacillus acidophilus*, *Streptococcus thermophiles*, *Lactobacillus casei*, *Bifidobacterium longum*, *Lactobacillus bulgaricus*, *Bifidobacterium breve*, *Lactobacillus rhamnosus*,	34.5 years (mean)	1 × 10^9^ CFU/g	80 days	Significantly meliorated normal morphology, motility, sperm concentration, DNA fragmentation, and sperm lipid peroxidation.	[216]
*Bifidobacterium longum*, *Streptococcus thermophilus*, *Lactobacillus rhamnosus*, *Lactobacillus acidophilus*, *Bifidobacterium breve*, *Lactobacillus casei*, *Lactobacillus bulgaricus*,	≥ 18 years	1 × 10^9^ CFU/capsule	90 days	Significantly improved sperm concentration and normal morphology and slightly improved sperm motility and volume after varicocelectomy, demonstrating the benefits of probiotics in improving semen parameters.	[217]
*Lactobacillus rhamnosus* MG4288, *Lactobacillus fermentum* MG901, *Lactobacillus paracasei* MG4272, *Lactobacillus plantarum* MG989, *Lactobacillus salivarius* MG242,	19–50 years	1.0 × 10^9^ CFU/g each	84 days	Significant reduction in Nugent score, a substantial increase in *Lactobacillus plantarum* in the vagina, and suppression in pathogenic bacteria such as *Atopobium vaginae*, *Gardnerella vaginalis*, and *Mobiluncus* spp., exhibiting bacterial vaginosis (BV) alleviation.	[218]
*Lactobacillus acidophilus* LA-5	18–49 years	1 × 10^9^ CFU/g	65 days	*Lactobacillusacidophilus* supplementation in treating vulvovaginal candidiasis (VVC) and decreasing negative culture, vulvovaginal erythema, and abnormal discharge, dyspareunia, dysuria, frequent urination, and burning is enormously comparable to fluconazole.	[219]
*Lactobacillus fermentum* LF26, *Lactobacillus delbrueckii* subsp. *lactis* LDL114, *Lactobacillus rhamnosus* LRH10, *Lactobacillus plantarum* LP115, *Lactobacillus. paracasei* LPC12, *Lactobacillus. helveticus* LA25	28.95 ± 0.70 years	3.2 × 10^9^ CFU	56 days	Significantly reduced discharge, burning, and irritation, along with reduced vulvovaginal symptoms, recurrences of VC, recurrences of social and emotional stress, exhibiting the alleviation of disease and increased defecation times per week, showing a reduced risk of pregnancy-induced constipation.	[220]
*Lactobacillus crispatus* DSM32716, DSM32717, DSM32718, DSM32720,	18–50 years	3 × 10^10^ CFU/capsule	90 days	Significantly reduced symptoms of BV and VVC, remarkable improvements in the smell and amount of discharge, Nugent score, and irritation/itching. Significantly improved vaginal lactobacilli counts and decreased BV-related bacteria.	[221]
Probiotics Alleviate Neurodegenerative and Neurodevelopmental Disorders					
*Lactobacillus plantarum* P8	31.7 ± 11.1 years	1 × 10^10^ CFU/g	84 days	Significantly reduced stress and anxiety. Slightly altered plasma cortisol levels and significantly reduced TNF-α and IFN-γ. Improved cognitive and memory traits, including verbal learning and memory and social-emotional cognition, in stressed adults.	[222]
*Bifidobacterium longum* R0175 or *Lactobacillus rhamnosus* HA-114	50–90 years	1 × 10^5^ CFU/g	84 days	Significant improvement in cognition in patients with Alzheimer’s disease.	[223]
*Bifidobacterium breve*, *Bifidobacterium infantis*, *Lactobacillus* *plantarum*, *Lactobacillus acidophilus*, *Streptococcus thermophilus*, *Lactobacillus casei*, *Bifidobacterium longum*, *Lactobacillus delbrueckii* *subsp. bulgaricus*	3–12 years	9 × 10^9^ CFU/g	56 days	Significantly improved pediatric quality of life inventory (PedsQL) and GI complaints in children on the autism spectrum.	[224]
*Lactococcus lactis W58*, *Lactococcus lactis W19*, *Bifidobacterium lactis W51* and *W52*, *Bifidobacterium bifidum W23*, *Lactobacillus brevis W63*, *Lactobacillus acidophilus W37*, *Lactobacillus salivarius W24*, *Lactobacillus casei W5*	~21 years	2.5 × 10^9^ CFU/g	28 days	Significant improvement in memory performance, attributed to the neural changes in the frontal cortex occurred during cognitive control interventions. Implemented measures to mitigate the adverse impacts of stress on cognitive function.	[225]
*Bifidobacterium longum* BIA-8, *Bifidobacterium lactis* BIA-7, *Bifidobacterium bifidum* BIA-6, *Lactobacillus acidophilus* T16	6.64 ± 10.69 years	2.7 × 10^7^ CFU/g each	84 days	Significantly decreased hospital anxiety and depression scale (HADS) depression scores, along with significant changes in HADS-ANX scores. Significantly increased the serum brain-derived neurotrophic factor in patients with depression.	[226]
*Lactobacillus casei* Shirota	19–22 years	3 × 10^10^ CFU/g	42 days	Significant decrease in stress and anxiety levels of participants and a significant improvement in their aerobic capacities.	[227]
